# Efficacy and safety of various immunosuppressive agents for pediatric Henoch-Schönlein purpura nephritis (IgA vasculitis nephritis) in Chinese children: a systematic review and network meta-analysis

**DOI:** 10.3389/fimmu.2026.1868631

**Published:** 2026-07-31

**Authors:** Tianjiao Jin, Shuo Yang, Yanlu Cai, Haokai Wang, Hongpeng Li, Keying Xu, Dan Sun, Huanmin Li, Xinmin Li

**Affiliations:** 1 First Teaching Hospital of Tianjin University of Traditional Chinese Medicine, Tianjin, China; 2 National Clinical Research Center for Chinese Medicine Acupuncture and Moxibustion, Tianjin, China

**Keywords:** Henoch-Schönlein Purpura nephritis, IgA vasculitis nephritis, immunosuppressive agents, meta-analysis, pediatric, systematic review

## Abstract

**Background:**

Henoch-Schönlein purpura nephritis (HSPN) is a common secondary glomerular disease in children. Approximately 30% of children with Henoch-Schönlein purpura develop this renal complication, and among them, about 20% may progress to chronic kidney disease. Current treatment relies on glucocorticoids combined with immunosuppressive agents. However, direct comparisons between different immunosuppressive regimens are lacking, and the optimal clinical approach remains undefined. Given that the efficacy of certain agents, such as mycophenolate mofetil, may vary across ethnic populations, this network meta-analysis (NMA) was conducted to evaluate the comparative efficacy and safety of immunosuppressive regimens specifically within the Chinese pediatric population with HSPN.

**Methodology:**

We systematically searched English and Chinese databases, including PubMed, Cochrane Library, Embase, Web of Science, China National Knowledge Infrastructure, Wanfang Academic Journal Full Text Database, and China Biomedical Literature Database to identify randomized controlled trials (RCTs) that evaluated immunosuppressive therapies for pediatric Henoch-Schönlein purpura nephritis (IgA vasculitis nephritis). Network meta-analysis was performed using Stata 18.0, and the quality of the included studies along with the risk of bias was assessed using RevMan 5.4.1.

**Results:**

Among the core efficacy outcomes, mycophenolate mofetil combined with conventional therapy (MMF+CT) ranked highest in reducing 24-hour urine protein (SUCRA = 80.4%) and elevating serum albumin (SUCRA = 86.0%). Tacrolimus combined with conventional therapy (TAC+CT) performed best in improving serum creatinine levels (SUCRA = 97.0%). Tripterygium wilfordii polyglycoside combined with conventional therapy (TWP+CT) was the most effective in shortening the time to resolution of hematuria (SUCRA = 98.7%) and proteinuria (SUCRA = 100.0%). In terms of safety, cyclosporine A combined with conventional therapy (CsA+CT) demonstrated the lowest risk of adverse events (SUCRA = 85.2%). Cluster analysis further indicated that mycophenolate mofetil combined with conventional therapy (MMF+CT) offered the most favorable balance between overall efficacy and safety.

**Conclusions:**

Immunosuppressive agents have significantly improved treatment outcomes for Chinese children with HSPN. Mycophenolate mofetil combined with conventional therapy (MMF+CT) offers the best balance between efficacy and safety, particularly for controlling proteinuria and hypoalbuminemia. Tacrolimus combined with conventional therapy (TAC+CT) is more suitable for patients with impaired renal function, whereas Tripterygium wilfordii polyglycoside combined with conventional therapy (TWP+CT) is effective for rapid symptom control. These findings support a shift toward individualized, phenotype-driven treatment strategies for this population. However, the results are predominantly derived from Chinese RCTs and may not be generalizable to other ethnic groups. Future research should prioritize large-scale, multicenter, long-term RCTs in diverse populations to verify the long-term stability of these findings and standardize adverse event reporting criteria.

**Systematic Review Registration:**

https://www.crd.york.ac.uk/PROSPERO/, identifier CRD420251234454.

## 1 Introduction

Henoch-Schönlein purpura (HSP) is an IgA-dominant small-vessel vasculitis that manifests clinically with skin purpura, arthralgia, abdominal pain, and renal involvement (1). In pediatric populations, approximately 30% of patients with HSP develop nephritis, termed Henoch-Schönlein purpura nephritis (HSPN), with around 20% of these cases potentially advancing to chronic kidney disease (2). The pathognomonic feature of HSPN is the deposition of immune complexes, notably abnormally glycosylated IgA1, within the glomeruli. These aberrant IgA1 molecules are targeted by anti-glycan antibodies, resulting in the formation of circulating immune complexes that deposit in the glomeruli and precipitate renal injury (3, 4). Dysregulation of both cellular and humoral immunity is pivotal in the immunopathogenesis of HSPN. Research indicates that the activation of cytotoxic T lymphocytes and natural killer cells in the peripheral blood of HSPN patients correlates with the onset of nephritis, potentially mediated through the interactions between CX3CL1 and CX3CR1 (5). Moreover, alterations in T-cell subsets have been documented in Henoch-Schönlein purpura nephritis (HSPN), characterized by increased proportions of effector memory CD8+ T cells and Th17 cells, along with decreased proportions of regulatory T cells and Th1 cells. These immunological changes may be associated with the varied clinical manifestations of the disease (6). The current management strategy for HSPN primarily involves the use of glucocorticoids and immunosuppressive agents. Although glucocorticoids are extensively employed to mitigate inflammation and manage symptoms, their prolonged use can result in significant adverse effects (7, 8). Consequently, combination therapy with immunosuppressants such as cyclosporine A, tacrolimus, and mycophenolate mofetil has shown enhanced clinical efficacy and is linked to improved renal survival rates in long-term follow-up (9, 10). Other immunosuppressants, have also demonstrated therapeutic benefits in certain refractory cases (8, 11). Although immunosuppressive agents have demonstrated advantages in the treatment of Henoch-Schönlein purpura nephritis (HSPN), there is a notable absence of direct comparative studies to determine the optimal clinical regimen. To address this gap, the present study utilized a network meta-analysis (NMA) to systematically assess available randomized controlled trials. The NMA methodology facilitates an integrated comparison of multiple treatment regimens in terms of their relative efficacy and safety, thereby providing more robust evidence to inform clinical practice. This approach not only aids in identifying the most effective treatment options but also guides the design of future clinical trials. By integrating direct and indirect evidence, we aimed to determine the optimal therapeutic strategies for pediatric HSPN, providing robust, phenotype-driven evidence to guide clinical decision-making.

## 2 Methods

### 2.1 Study design and registration

The study selection process adhered to the guidelines of the Preferred Reporting Items for Systematic Reviews and Meta-Analyses (PRISMA) (12), and the meta-analysis protocol has been registered in Prospero (No.CRD420251234454).

### 2.2 Inclusion and exclusion criteria

Our inclusion and exclusion criteria were established in accordance with the PICOS framework. The PICOS principle is presented in **Table 1**.

**Table 1:** PICOS criteria.

Criteria	Explanation
Population	Children (≤18 years) diagnosed with Henoch-Schönlein purpura nephritis (IgA vasculitis with renal involvement)
Intervention	Any immunosuppressive regimen (e.g., MMF, TAC, CTX, LEF, CsA, TWP, alone or combined with conventional therapy)
Comparator	Other immunosuppressive agents or conventional supportive therapy (including ACEIs/ARBs, antiplatelet/anticoagulant agents, and corticosteroids)
Outcomes	Primary: 24-hour urinary protein quantification(24hU-Pro).Secondary: Incidence of adverse events (AE incidence), serum albumin (ALB) level, serum creatinine (SCr) level, blood urea nitrogen (BUN) level, and the time to resolution of proteinuria and/or hematuria(TTPR/TTHR)
Study Design	(RCTs)

Inclusion criteria: (1) Participants: Pediatric patients aged 18 years or younger, clinically diagnosed with Henoch-Schönlein purpura nephritis, also referred to as IgA vasculitis nephritis (IgAVN). (2) Diagnostic Criteria: Eligibility required adherence to the diagnostic criteria for pediatric Henoch-Schönlein purpura nephritis, as specified in the “Evidence-based Guidelines for the Diagnosis and Treatment of Henoch-Schönlein Purpura Nephritis (2016).” (13) This included the presence of hematuria and/or proteinuria manifesting within six months following the onset of Henoch-Schönlein purpura. (3) Interventions: The experimental cohort received at least one immunosuppressant, such as mycophenolate mofetil, cyclophosphamide, or tacrolimus, either as monotherapy or in conjunction with conventional therapy (CT). The control cohort was administered CT, a placebo, or an alternative immunosuppressant. In this study, conventional therapy (CT) was defined as glucocorticoid treatment. Although this definition also allows for adjunctive therapies such as ACE inhibitors or ARBs, it should be noted that in the included studies, the control groups uniformly received glucocorticoids as the core regimen, while the use of ACEIs or ARBs was rarely reported and, when present, was non-standardized. Therefore, in the context of our network meta-analysis, “CT” refers essentially to a glucocorticoid-based regimen. (4) Outcomes: Included studies were required to report at least one of the following: Primary outcome: 24-hour urinary protein quantification (24hU-Pro). Secondary outcomes: Incidence of adverse events (AE incidence), serum albumin (ALB) level, serum creatinine (SCr) level, blood urea nitrogen (BUN) level, and the time to resolution of proteinuria and/or hematuria(TTPR/TTHR). (5) Study Design: Randomized controlled trials (RCTs).

Exclusion criteria: (1) duplicate publications, where only the version with the most complete data or the earliest publication date was included; (2) studies for which the full text was unavailable, or which had incomplete data precluding the extraction of required outcome measures; (3) non-randomized study designs, including animal studies, meta-analyses/reviews, conference abstracts, letters to the editor/replies, guidelines, or case reports; (4) studies involving adult populations (age >18 years) or mixed populations (adults and children) from which pediatric data could not be extracted separately; (5) studies lacking an immunosuppressive agent as part of the intervention. Two reviewers independently screened the records, with any disagreements resolved through discussion and consensus with a third reviewer.

### 2.3 Search strategy

We conducted a comprehensive literature search using both automated and manual methods across several databases, including PubMed, Cochrane Library, Embase, Web of Science, China National Knowledge Infrastructure, Wanfang Academic Journal Full Text Database, and China Biomedical Literature Database, to supplement existing research. The deadline was November 30th, 2025. Study selection and data extraction were performed independently by two reviewers (Li HP and Xu KY). Any discrepancies were resolved through consultation with a third reviewer (Cai YL). The databases were chosen to prioritize those commonly utilized in contemporary meta-analyses and to encompass both English and Chinese randomized controlled trials relevant to the topic. In addition, an exhaustive search of existing meta-analyses and review articles was conducted to ensure the literature inclusion was as comprehensive as possible. Initially, the titles and abstracts of identified records were screened to exclude irrelevant studies. Subsequently, the full texts of the remaining articles were assessed for eligibility. Detailed search strategies are provided in **Supplementary Methods 1**.

### 2.4 Data extraction and quality assessment

Data extraction and quality assessment were independently performed by two reviewers (Li HP and Xu KY). Any disagreements were resolved through adjudication by a third reviewer (Cai YL). After extraction, the data underwent cross-checking; any remaining discrepancies were referred to the third reviewer for a final decision. The following information was extracted from each study: first author’s name, patient age and sex, year of disease onset as reported in the study, study design, and values of the outcome measures. All data were derived from the final outcome assessments.

The risk of bias for the included RCTs was assessed using the Cochrane Risk of Bias tool (version 5.1.0) (12). This tool evaluates five domains: allocation concealment; blinding of participants and personnel; blinding of outcome assessment; incomplete outcome data; and selective reporting. For each domain, the risk of bias was judged as “low risk,” “high risk,” or “some concerns.”

### 2.5 Statistical analysis

This study employed the mvmeta package in Stata 18.0 software for network and routine meta- analyses, using mean ± standard deviation as effect size metrics for quantitative data analysis. Literature quality and bias risk were evaluated via RevMan 5.4.1 software, while heatmaps were generated with R 4.3.1. All effect indicators were presented with 95% confidence intervals (CI). Heterogeneity was assessed using prediction interval plots, and local inconsistency was detected via node splitting methods. Clinical and methodological features were compared to evaluate evidence network similarity, with outcome comparisons between any two interventions presented in ranking tables. The significance level was set at α = 0.05. Consistency between direct and indirect comparisons was evaluated using an inconsistency test within closed loops. Consistency was indicated if the 95% confidence interval (CI) of the inconsistency factor (IF) included 0; an IF > 1 suggested the presence of loop inconsistency, which should be further examined using traditional pairwise meta-analysis to identify sources of heterogeneity. The surface under the cumulative ranking curve (SUCRA) values, ranging from 0% to 100%, were used to probabilistically rank the treatment effects, with higher values indicating a greater likelihood of being the optimal treatment.

## 3 Results

### 3.1 Literature search details

**Figure 1:**
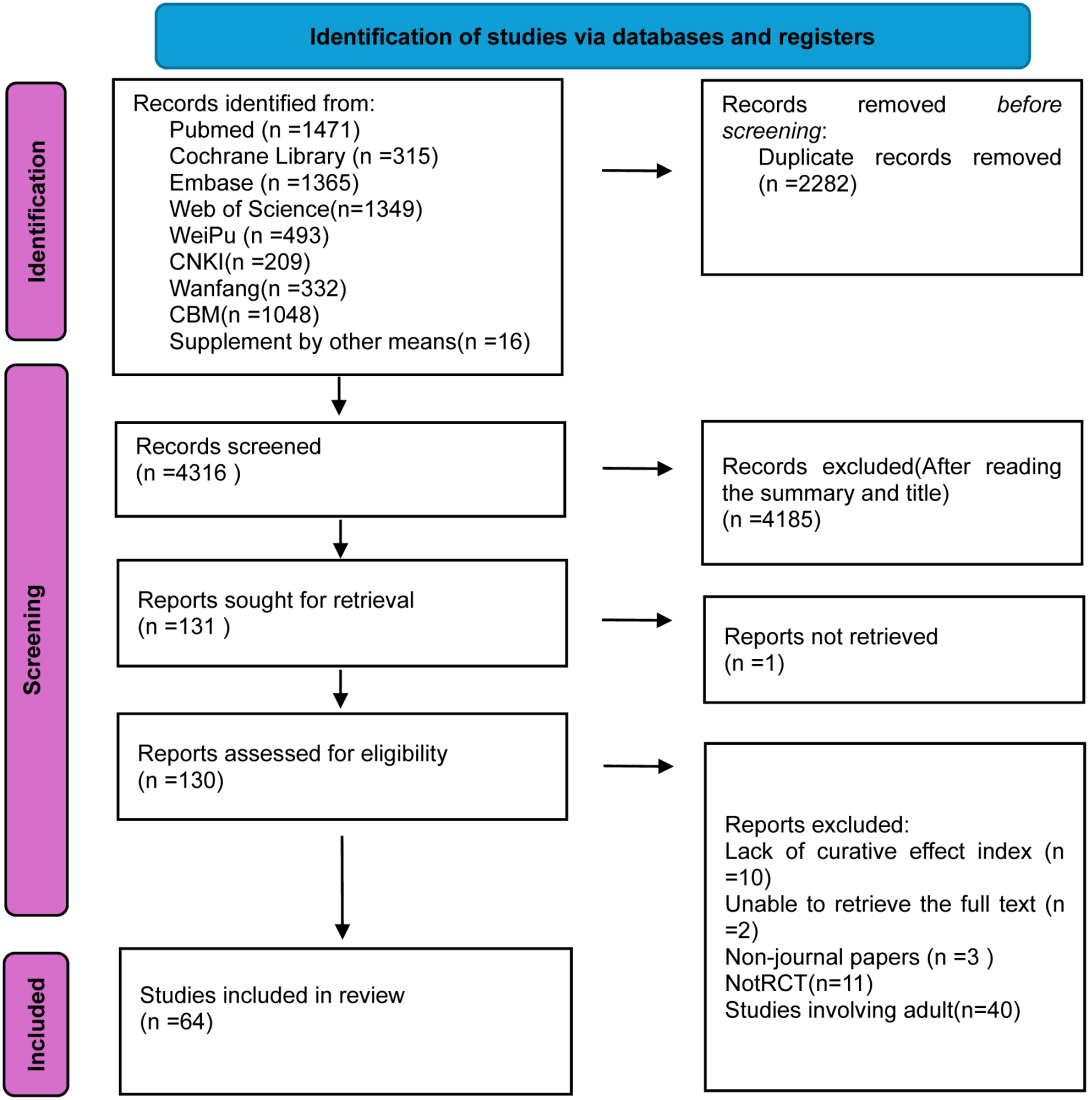
Document screening process and results.

**Figure 1** illustrates the literature screening process. Initially, a total of 6,598 studies were retrieved from the database. Duplicate articles and studies that did not satisfy the inclusion criteria were subsequently excluded. Following a comprehensive review of the texts, 64 articles were deemed to meet the inclusion criteria and were incorporated into the meta-analysis. Of these, 63 articles were in Chinese and 1 were in English, collectively involving 4,931 participants. The publication years of these studies range from 2005 to 2024. For a detailed account of the search terms employed, please consult the **Supplementary Methods 1**.

### 3.2 Study characteristics classification

A total of 64 (14–77) eligible RCTs met the inclusion criteria for this systematic review, involving a total of 4,931 participants. Of these, 58 RCTs provided extractable outcome data and were included in the quantitative network meta-analysis. Nine distinct intervention regimens were evaluated: cyclophosphamide (CTX), cyclophosphamide combined with CT (CTX+CT), tacrolimus (TAC), tacrolimus combined with CT (TAC+CT), mycophenolate mofetil combined with CT (MMF+CT), leflunomide combined with CT (LEF+CT), Tripterygium wilfordii polyglycoside combined with CT (TWP+CT), cyclosporine A combined with CT (CsA+CT), and tacrolimus combined with Tripterygium wilfordii polyglycoside (TAC+TWP). The study designs comprised one three-arm trial and 63 two-arm parallel-group trials. The basic characteristics of the included studies are detailed in **Supplementary Table 2**.

### 3.3 Literature quality evaluation

**Figure 2:**
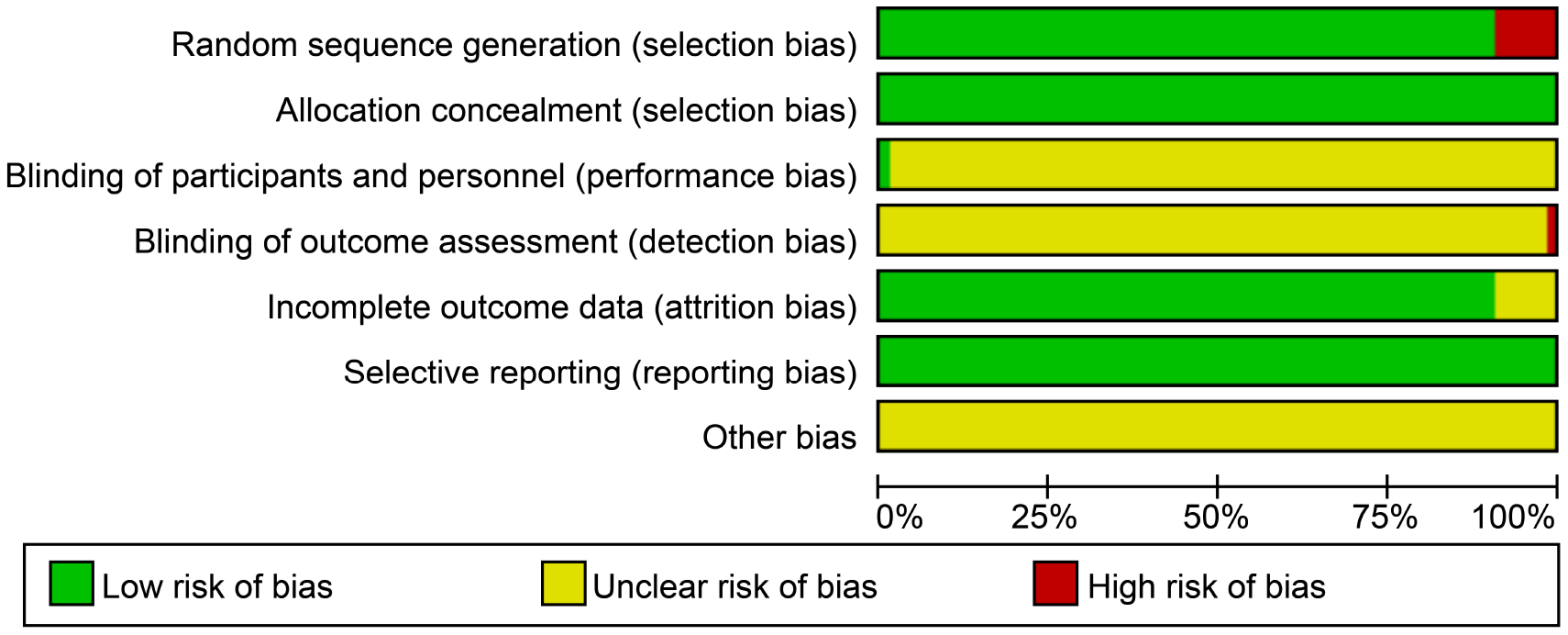
Risk of bias assessment included in the study.

Among the included studies, all 64 reported baseline comparability between groups. Specifically, 58 studies (90.6%) adequately described their randomization methods and were rated as low risk, while 6 studies (9.4%) (52, 59, 65, 75–77) did not clearly specify the randomization procedure and were thus deemed high risk. Blinding was not implemented in 63 studies (98.4%), resulting in an unclear risk classification (some concerns). One study (1.6%) (65) reported blinding of participants only and was rated as low risk for participant blinding but high risk for blinding of outcome assessment. Complete outcome data were provided in 58 studies (90.6%), which were assessed as low risk and were included in the quantitative NMA; the remaining 6 studies (9.4%) (18, 25, 30, 37, 49, 73) had incomplete data and were therefore included only in the qualitative synthesis. Allocation concealment was rated as low risk across all studies. To assess selective reporting, we cross-verified the consistency between the methods and results sections of each study; all were found to be at low risk with fully reported outcomes. However, other potential sources of bias remained unclear. The risk of bias assessment for the 64 included studies is summarized in **Figure 2**, with detailed results available in **Supplementary Figure 1**.

## 4 Network meta-analysis

**Figure 3:**
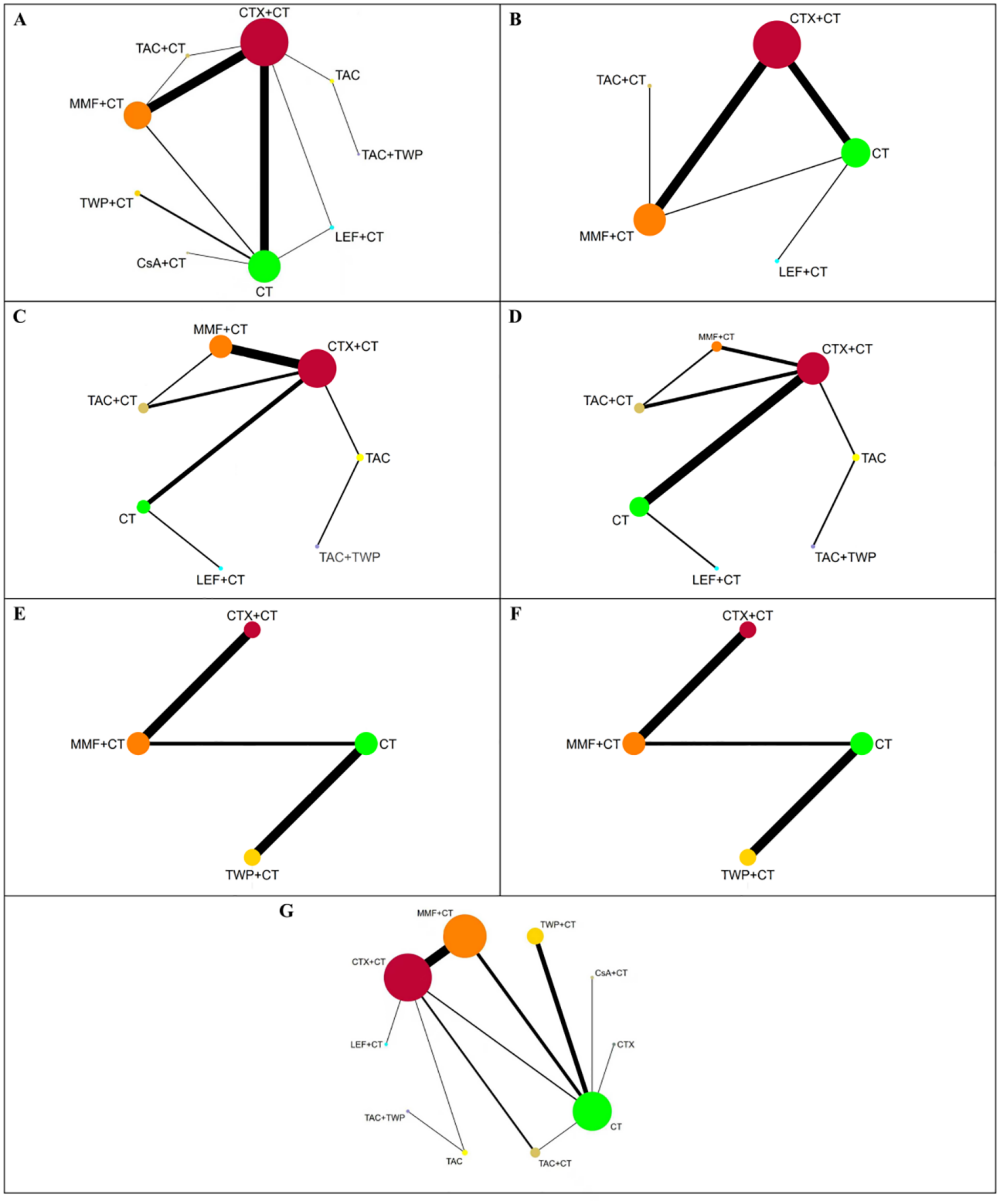
The evidence network of all papers on different treatments. **(A)** 24hU-Pro; **(B)** ALB; **(C)** SCr; **(D)** BUN; **(E)** TTPR; **(F)** TTHR; **(G)** AE incidence. The thickness of the lines represents the number of studies, and the sizes of the nodes indicate the total sample sizes for each treatment. 24hU-Pro:24-Hour Urine Protein Quantification; ALB: Serum albumin; SCr: Serum creatinine; BUN, Blood urea nitrogen; TTPR, Time To Proteinuria Resolution; TTHR, Time To Hematuria Resolution; AE incidence, Incidence of adverse events.

The 64 included studies evaluated 9 distinct interventions. **Figure 3** presents the network structure of all treatments for each outcome. The thickness of the lines (edges) corresponds to the number of studies providing direct head-to-head comparisons between two interventions, while the diameter of each node is proportional to the total sample size of the corresponding treatment group.

### 4.1 24hU-Pro

A total of 38 randomized controlled trials, involving 2,779 patients, reported 24-hour urine protein (24hU-Pro) levels and evaluated 8 different immunosuppressive treatment regimens. The network of comparisons among these interventions is illustrated in **Figure 3A**. Since the test for inconsistency yielded P > 0.05, a consistency model was deemed appropriate for the network meta-analysis. The analysis revealed that, regarding 24-hour urine protein levels, five different immunosuppressive regimens showed statistically significant superiority over conventional therapy or placebo. Specifically, MMF+CT [MD = −1.60 g/24h, 95% CI (−2.06, −1.14)], LEF+CT [MD = −1.59 g/24h, 95% CI (−2.91, −0.26)], TAC+CT [MD = −1.36 g/24h, 95% CI (−2.28, −0.44)], and TAC alone [MD = −1.31 g/24h, 95% CI (−2.60, −0.02)] all demonstrated a significant reduction in 24hUP levels (all p < 0.05). Furthermore, MMF+CT was significantly more effective in reducing 24hUP levels than both CTX+CT [MD = −0.35 g/24h, 95% CI (−0.69, −0.01)] and TWP+CT [MD = −1.43 g/24h, 95% CI (−2.27, −0.59)]. Notably, compared to the TWP+CT group, both TAC+CT [MD = −1.19 g/24h, 95% CI (−2.35, −0.03)] and CTX+CT [MD = −1.08 g/24h, 95% CI (−1.88, −0.29)] showed better therapeutic effects. No statistically significant differences were observed in other pairwise comparisons (p > 0.05). Detailed results are provided in **Supplementary Table 3**.

**Figure 4:**
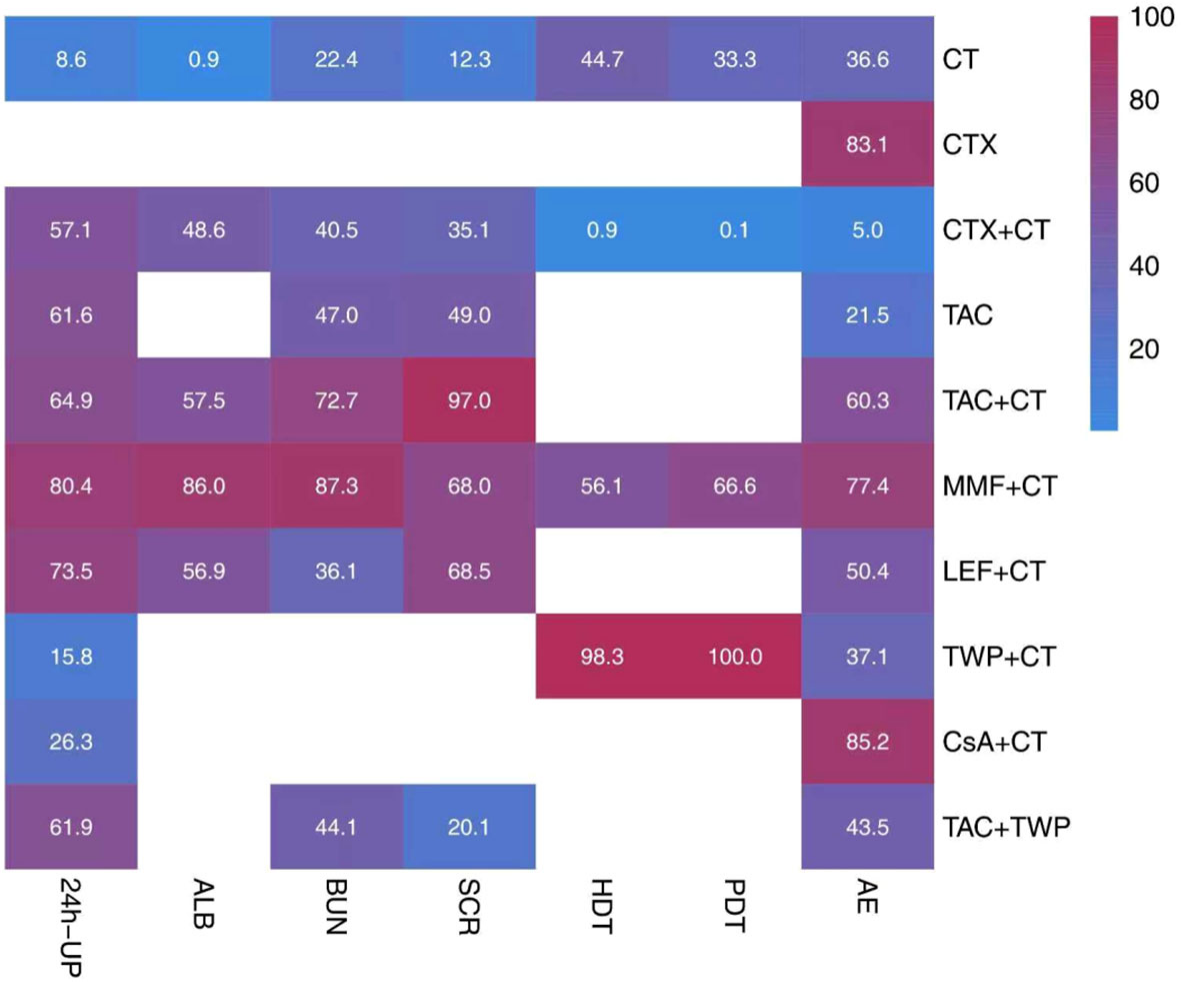
Heat map of SUCRA values for various intervention measures. The SUCRA values, ranging from 0 to 100%, indicate the likelihood of each intervention being the most effective treatment. 24hU-Pro:24-Hour Urine Protein Quantification; ALB, Serum albumin; SCr, Serum creatinine; BUN, Blood urea nitrogen; TTPR, Time To Proteinuria Resolution; TTHR, Time To Hematuria Resolution; AE incidence, Incidence of adverse events.

**Figure 5:**
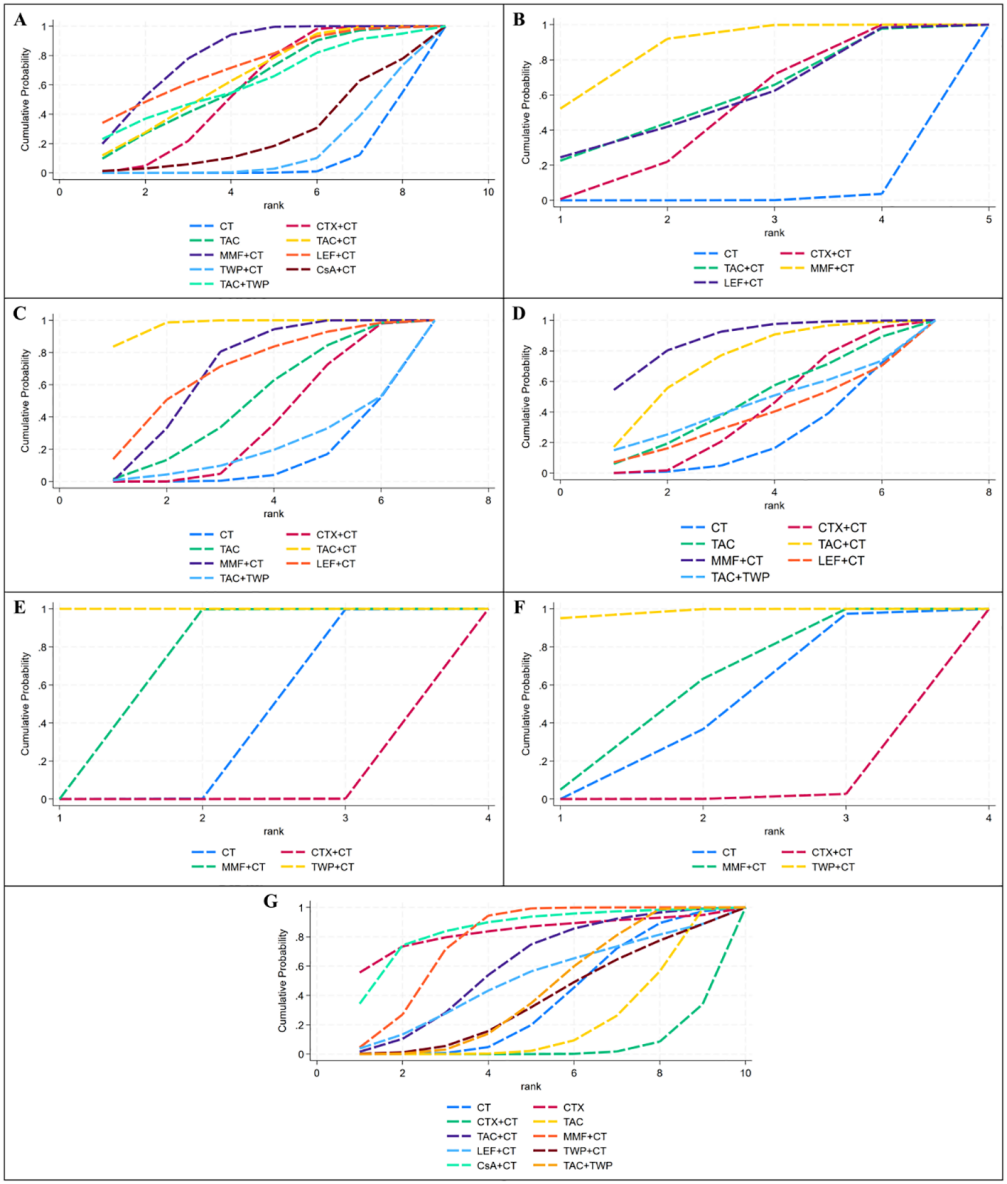
The SUCRA values for Principal Outcomes. **(A)** 24hU-Pro; **(B)** ALB; **(C)** SCr; **(D)** BUN; **(E)** TTPR; **(F)** TTHR; **(G)** AE incidence. The thickness of the lines represents the number of studies, and the sizes of the nodes indicate the total sample sizes for each treatment. 24hU-Pro:24-Hour Urine Protein Quantification; ALB, Serum albumin; SCr, Serum creatinine; BUN, Blood urea nitrogen; TTPR, Time To Proteinuria Resolution; TTHR, Time To Hematuria Resolution; AE incidence, Incidence of adverse events.

Surface under the cumulative ranking curve (SUCRA) analysis indicated that MMF+CT (80.4%) and LEF+CT (73.5%) were ranked as the most effective interventions for improving 24hU-Pro levels. The complete SUCRA values and intervention rankings for all outcomes are detailed in **Supplementary Table 10** and illustrated in **Figures 4** and **5A**.

Closed loops formed among the interventions in this study, and loop inconsistency tests were conducted. Loop inconsistency refers to a discrepancy between direct and indirect evidence within a closed loop of treatment comparisons in a network meta-analysis, which may affect the reliability of conclusions and is assessed using the inconsistency factor (IF). An IF value exceeding 1 suggests the presence of loop inconsistency. The tests revealed no statistically significant inconsistency in two loops: the loop formed by CT, CTX+CT, and LEF+CT [IF = 1.60, 95% CI (0, 4.64), p = 0.30] and the loop formed by CTX+CT, TAC+CT, and MMF+CT [IF = 0.12, 95% CI (0.00, 0.65), p = 0.66], as their p-values were greater than 0.05. However, statistically significant inconsistency was detected in the loop formed by CT, CTX+CT, and MMF+CT [IF = 0.91, 95% CI (0.14, 1.68), p = 0.02], where the 95% confidence interval for the IF did not include zero and the p-value was less than 0.05. These results are summarized in **Table 2**.

**Table 2:** Evaluation of local inconsistency within treatment loops.

Outcome	Loop	IF	95%CI	P
24hU-Pro	CT-CTX+CT-LEF+CT	1.6	[0,4.64]	0.3
	CT-CTX+CT-MMF+CT	0.91	[0.14,1.68]	0.02
	CTX+CT-TAC+CT-MMF+CT	0.12	[0,0.65]	0.66
ALB	CT-CTX+CT-MMF+CT	0.852	[0,2.02]	0.153
BUN	CTX+CT-TAC+CT-MMF+CT	3.18	[1.15,5.22]	0.002
SCr	CTX+CT-TAC+CT-MMF+CT	22.16	[11.74,32.58]	< 0.001
AE incidence	CT-CTX+CT-TAC+CT	1.54	[0,3.38]	0.099
	CT-CTX+CT-MMF+CT	0.2	[0,1.73]	0.041

24hU-Pro, 24-Hour Urine Protein Quantification; ALB, Serum albumin; BUN, Blood urea nitrogen; SCr, Serum creatinine; AE incidence, Incidence of adverse events.

### 4.2 ALB

A total of 18 randomized controlled trials, involving 1,246 patients, reported serum albumin (ALB) levels and evaluated 4 different immunosuppressive treatment regimens. The network of comparisons among these interventions is illustrated in **Figure 3B**, and a consistency model was applied for the network meta-analysis. The NMA demonstrated that, compared to the CT group, all immunosuppressive regimens showed statistically significant efficacy in increasing serum albumin levels: MMF+CT [SMD = 1.07, 95% CI (0.69, 1.45)], TAC+CT [SMD = 0.84, 95% CI (0.01, 1.66)], LEF+CT [SMD = 0.82, 95% CI (0.04, 1.59)], and CTX+CT [SMD = 0.78, 95% CI (0.48, 1.08)] (all p < 0.05), with MMF+CT showing the most pronounced effect. Furthermore, MMF+CT was significantly more effective than CTX+CT [SMD = 0.29, 95% CI (0.03, 0.54)]. However, no statistically significant differences were observed in the pairwise comparisons among MMF+CT, LEF+CT, and TAC+CT regarding their effect on serum albumin elevation. Detailed pairwise comparison results are provided in **Supplementary Table 4**.

SUCRA analysis indicated that MMF+CT (86.0%) was ranked as the most effective intervention for improving ALB levels (**Supplementary Table 10**, **Figures 4**, **5B**).

Closed loops formed among the interventions, and consistency within these loops was tested. The loop inconsistency test for the closed loop formed by CT, CTX+CT, and MMF+CT [IF = 0.85, 95% CI (0, 2.02), P = 0.153] indicated no statistically significant discrepancy between direct and indirect evidence, suggesting minimal heterogeneity within this loop. These results are detailed in **Table 2**.

### 4.3 SCr

A total of 15 randomized controlled trials, involving 1,211 patients, reported serum creatinine (SCr) levels and evaluated 6 different immunosuppressive treatment regimens. The network of comparisons among these interventions is illustrated in **Figure 3C**, and a consistency model was applied for the network meta-analysis.

The NMA revealed that, for SCr levels, three different immunosuppressive regimens demonstrated statistically significant superiority over conventional therapy. Compared to the CT group, TAC+CT [SMD = −2.49, 95% CI (−3.67, −1.32)], LEF+CT [SMD = −1.49, 95% CI (−2.94, −0.05)], and MMF+CT [SMD = −1.36, 95% CI (−2.35, −0.37)] were significantly more effective in reducing SCr levels (all p < 0.05). Furthermore, TAC+CT was more effective than TAC alone [SMD = −1.63, 95% CI (−3.22, −0.04)], CTX+CT [SMD = −1.90, 95% CI (−2.73, −1.07)], and TAC+TWP [SMD = −2.40, 95% CI (−4.49, −0.31)] (all p < 0.05). Notably, MMF+CT was also more effective than CTX+CT [SMD = −0.77, 95% CI (−1.32, −0.22)] in lowering SCr levels. No other statistically significant differences were observed in the remaining pairwise comparisons (p > 0.05). Detailed results are provided in **Supplementary Table 5**.

Loop inconsistency was detected in the closed loop formed by CTX+CT, TAC+CT, and MMF+CT [IF = 22.16, 95% CI (11.74, 32.58), P = 0.00], indicating a statistically significant discrepancy between direct and indirect evidence. The large magnitude of the IF suggests a substantial inconsistency, which may originate from heterogeneity within the interventions forming the loop.

SUCRA analysis indicated that TAC+CT (97.0%) was ranked as the most effective intervention for improving SCr levels (**Supplementary Table 10**, **Figures 4**, **5C**).

### 4.4 BUN

A total of 13 randomized controlled trials, involving 995 patients, reported blood urea nitrogen (BUN) levels and evaluated 6 different immunosuppressive treatment regimens. The network of comparisons among these interventions is illustrated in **Figure 3D**, and a consistency model was applied for the network meta-analysis. Notably, the NMA indicated that MMF+CT significantly reduced BUN levels compared to several other treatments, including CT alone [MD = −2.53, 95% CI (−4.81, −0.25)] and CTX+CT [MD = −1.90, 95% CI (−3.75, −0.06)], with all differences being statistically significant (p < 0.05). No other statistically significant differences were observed in the pairwise comparisons between the remaining groups (p > 0.05). Detailed pairwise comparison results are available in **Supplementary Table 6**. SUCRA analysis indicated that MMF+CT (87.3%) and TAC+CT (72.7%) were ranked as the most effective interventions for improving BUN levels (**Supplementary Table 10**, **Figures 4**, **5D**).

Loop inconsistency was detected in the closed loop formed by CTX+CT, TAC+CT, and MMF+CT [IF = 3.18, 95% CI (1.15, 5.22), P = 0.002], suggesting a statistically significant discrepancy between direct and indirect evidence within this loop. These results are detailed in **Table 2**.

### 4.5 TTPR

A total of 12 randomized controlled trials, involving 935 patients, reported the time to proteinuria resolution (TTPR) and evaluated 3 different immunosuppressive treatment regimens. The network of comparisons among these interventions is illustrated in **Figure 3E**, and a consistency model was applied for the network meta-analysis. Compared to the CT group, both TWP+CT [MD = -9.56 days, 95% CI (-17.73, -10.64)] and MMF+CT [MD = -3.77 days, 95% CI (-6.41, -1.13)] were significantly more effective in shortening the time to proteinuria resolution (all p < 0.05). Furthermore, TWP+CT [MD = -13.89 days, 95% CI (-17.41, -10.38)], MMF+CT [MD = -8.10 days, 95% CI (-9.67, -6.53)], and CT alone [MD = -4.33 days, 95% CI (-7.44, -1.23)] all demonstrated superior efficacy compared to the CTX+CT group. The 95% confidence intervals for all regimens did not include zero, indicating statistically significant results. Notably, the MMF+CT regimen had the narrowest confidence interval, suggesting greater precision and stability of its estimated effect. Detailed results are provided in **Supplementary Table 7**. SUCRA analysis indicated that TWP+CT (100.0%) was ranked as the most effective intervention for shortening the time to proteinuria resolution (**Supplementary Table 10**, **Figures 4**, **5E**).

### 4.6 TTHR

A total of 12 randomized controlled trials, involving 935 patients, reported the time to hematuria resolution (TTHR) and evaluated 3 different immunosuppressive treatment regimens. The network of comparisons among these interventions is illustrated in **Figure 3F**, and a consistency model was applied for the network meta-analysis. The NMA demonstrated that, compared to the CT group, TWP+CT [MD = −26.21 days, 95% CI (−40.23, −12.19)] was significantly more effective in shortening the time to hematuria resolution (p < 0.05). Furthermore, when compared to the CTX+CT group, both TWP+CT [MD = −52.17 days, 95% CI (−81.82, −22.53)] and MMF+CT [MD = −29.90 days, 95% CI (−43.93, −15.88)] resulted in a significantly shorter time to hematuria resolution (all p < 0.05). Detailed pairwise comparison results are provided in **Supplementary Table 8**. SUCRA analysis indicated that TWP+CT (98.7%) was ranked as the most effective intervention for reducing the time to hematuria resolution (**Supplementary Table 10**, **Figures 4**, **5F**).

### 4.8 AE incidence

Data on adverse events (AE incidence) were systematically extracted from all included randomized controlled trials (RCTs). The reported adverse reactions primarily included rash, gastrointestinal disturbances, decreased peripheral white blood cell count, infections, and impaired liver or renal function. This analysis included 36 RCTs involving 2,873 patients, which compared 10 different immunosuppressive treatment regimens. The relative risk (RR) with a 95% confidence interval (CI) was used as the effect measure. The network of comparisons is illustrated in **Figure 3G**, and a consistency model was applied for the network meta-analysis. Compared to the CT group, MMF+CT [RR = 0.55, 95% CI (0.34, 0.88)] was associated with a significantly lower risk of adverse events (P < 0.05). Furthermore, when compared to the CTX+CT group, several regimens showed a significantly reduced risk: MMF+CT [RR = 0.37, 95% CI (0.29, 0.47)], CsA+CT [RR = 0.22, 95% CI (0.06, 0.86)], TAC+CT [RR = 0.48, 95% CI (0.24, 0.95)], TAC alone [RR = 0.82, 95% CI (0.68, 0.99)], and TAC+TWP [RR = 0.64, 95% CI (0.46, 0.88)] (all P < 0.05). No other statistically significant differences were observed in the majority of pairwise comparisons between the remaining immunosuppressive regimens. Detailed results are provided in **Supplementary Table 9**. SUCRA analysis indicated that CsA+CT (85.2%) was associated with the lowest risk of adverse events, ranking it as the regimen with the optimal safety profile (**Supplementary Table 10**, **Figures 4**, **5G**).

Closed loops were formed among the interventions, and loop inconsistency tests were performed. The tests indicated no statistically significant inconsistency in two loops: the loop formed by CT, CTX+CT, and TAC+CT [IF = 1.54, 95% CI (0, 3.38), p = 0.10] and the loop formed by CT, CTX+CT, and MMF+CT [IF = 0.20, 95% CI (0.00, 1.73), p = 0.80], as their p-values were greater than 0.05. These results are detailed in **Table 2**.

Although the incidence of adverse events was generally low and mostly mild across all interventions, vigilance regarding safety data reporting remains necessary. Furthermore, it is evident that most included studies focused primarily on short-term safety, leading to a lack of comprehensive long-term safety data. Our analysis also revealed issues among the included studies, such as imprecise terminology, inconsistent implementation standards, and incomplete reporting. Future research should adhere to the CONSORT-Harms guidelines to provide detailed and comprehensive reporting on the potential risks of immunosuppressive agents.

### 4.9 Cluster analysis

**Figure 6:**
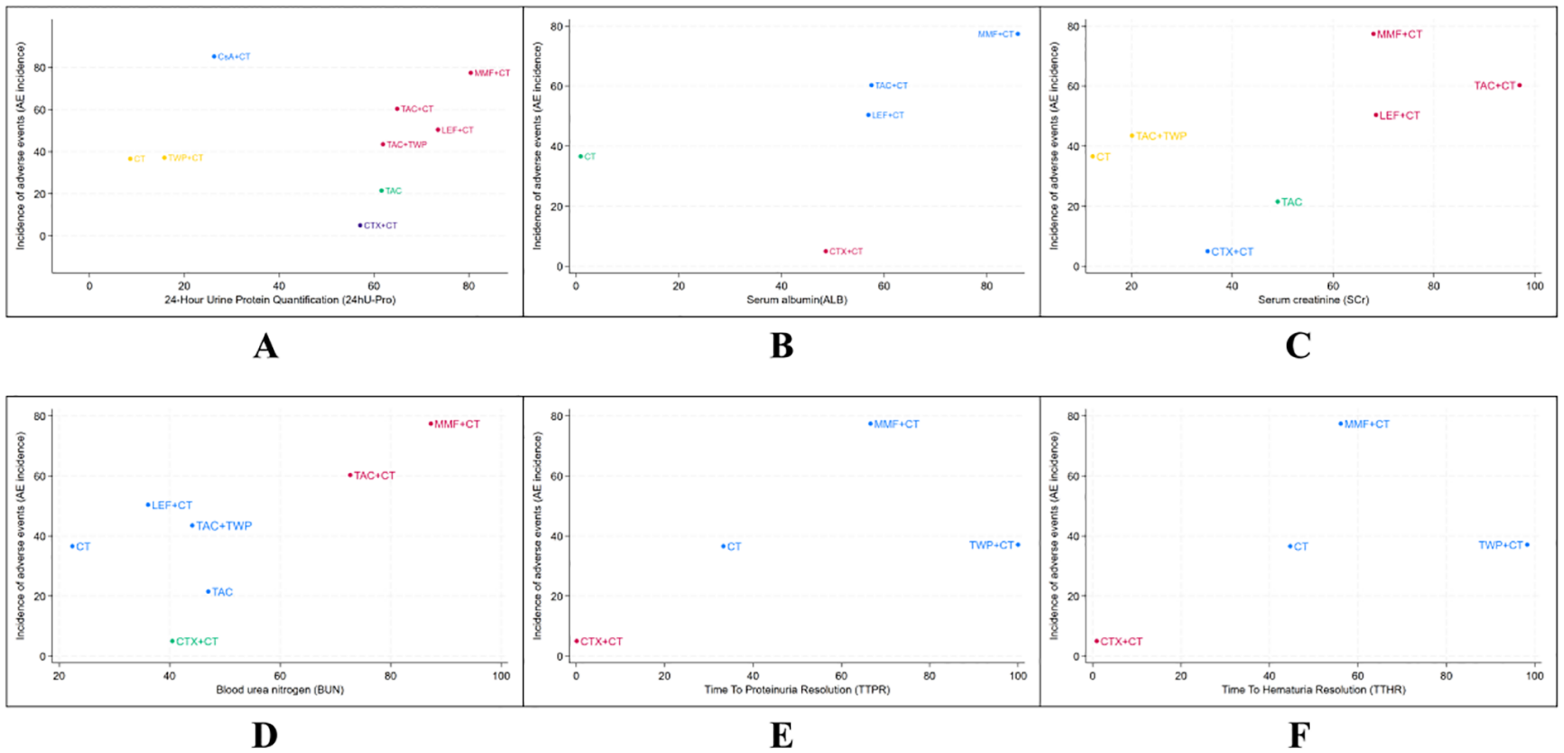
Clustering analysis plot for comparison between all outcomes and adverse events. **(A)** 24hU-Pro; **(B)** ALB; **(C)** SCr; **(D)** BUN; **(E)** TTPR; **(F)** TTHR. 24hU-Pro:24-hour urine protein quantification; AE incidence, Incidence of adverse events; ALB, Serum albumin; SCr, Serum creatinine; BUN, Blood urea nitrogen; TTPR, Time To Proteinuria Resolution; TTHR, Time To Hematuria Resolution.

Cluster analysis was performed to identify the most effective immunosuppressive treatment regimen by evaluating parameters including 24hU-Pro, AE incidence, ALB, SCr, BUN, TTPR, and TTHR. As shown in **Figure 6**, the results indicated that MMF+CT demonstrated a superior profile across multiple dimensions compared to other regimens and could be considered the best-performing option overall. Specifically, it achieved high scores for renal function indicators (BUN, SCr) alongside the highest safety score. For nutritional status, it yielded the highest ALB level, again paired with the top safety score. Regarding symptom resolution, while the time to proteinuria and hematuria resolution was not the shortest, it corresponded with the higher safety score. This pattern suggests that the MMF+CT regimen, potentially through its distinct immunosuppressive mechanism, achieves significant efficacy (reflected in favorable biochemical markers) while maintaining an optimal safety balance.

In contrast, the CTX+CT regimen presented a different profile: it achieved the fastest symptom resolution but scored in the low-to-mid range on several efficacy indicators (e.g., BUN, SCr, ALB). Its corresponding safety SUCRA score consistently ranked the lowest. This indicates that while this regimen may control symptoms rapidly, its safety profile is potentially less advantageous compared to other options.

The performance of the remaining regimens—CT alone, TAC alone, LEF+CT, and TAC+TWP—generally fell between the two patterns described above. For instance, CT alone showed moderate efficacy scores for BUN, TTPR, and TTHR, with a corresponding middle-range safety score. The TAC alone regimen maintained an above-average safety score while achieving a high efficacy score for 24hU-Pro. The LEF+CT and TAC+TWP regimens demonstrated higher efficacy values than CT alone for indicators like BUN, accompanied by correspondingly favorable safety scores.

Therefore, the cluster analysis results suggest that if the safety SUCRA score is prioritized as the primary criterion, MMF+CT emerges as the best-performing immunosuppressive combination. It exhibits a pattern of “high efficacy accompanied by high safety scores,” even though the speed of clinical symptom resolution is not the fastest. This finding provides a quantitative basis for clinical treatment selection, grounded in a comprehensive efficacy-safety profile.

### 4.10 Publication bias

**Figure 7:**
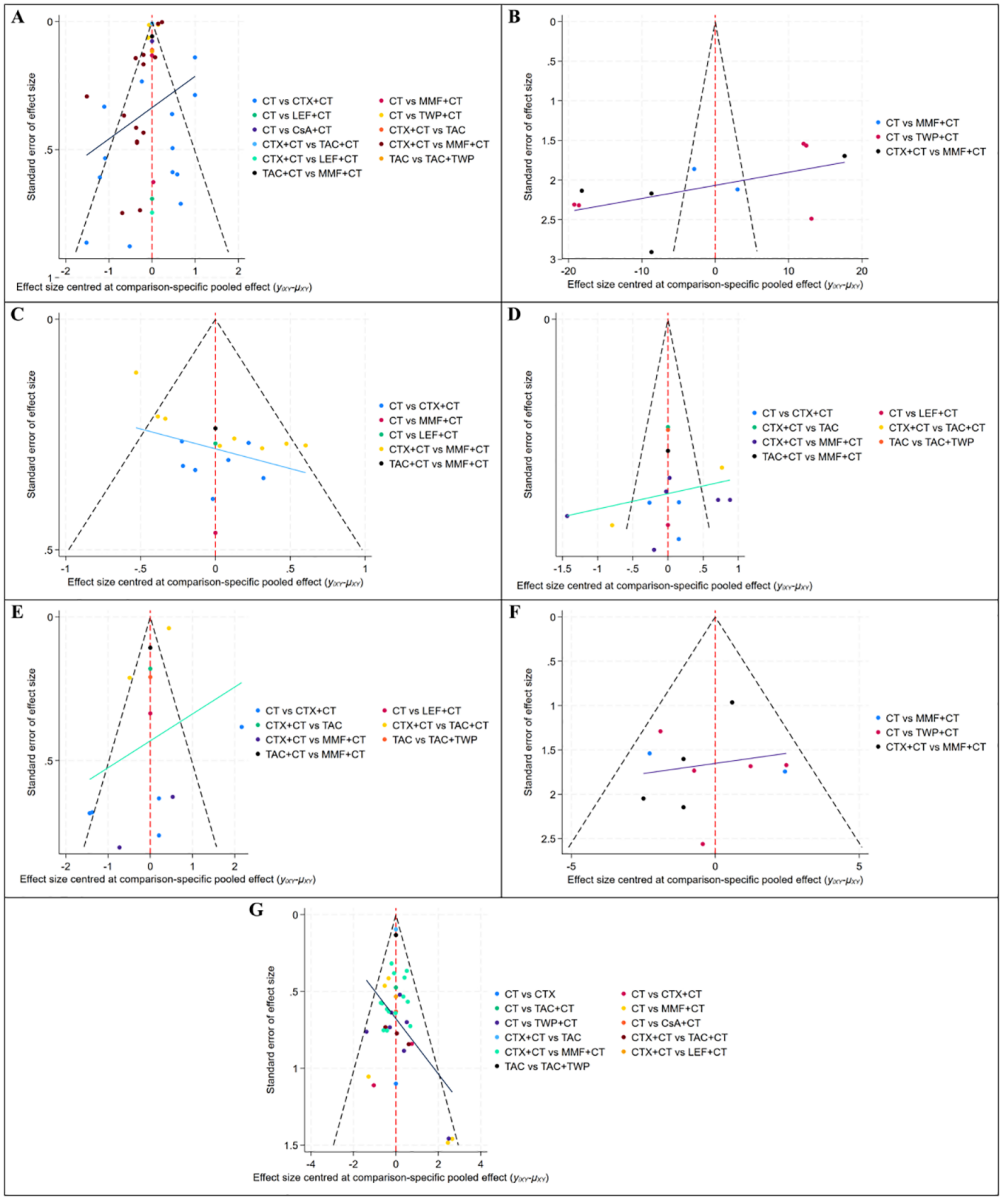
Funnel plot for publication bias in all outcomes. **(A)** 24hU-Pro; **(B)** ALB; **(C)** SCr; **(D)** BUN; **(E)** TTPR; **(F)** TTHR; **(G)** AE incidence. 24hU-Pro:24-Hour Urine Protein Quantification; ALB, Serum albumin; SCr, Serum creatinine; BUN, Blood urea nitrogen; TTPR, Time To Proteinuria Resolution; TTHR, Time To Hematuria Resolution; AE incidence, Incidence of adverse events.

Funnel plots adjusted for bias were generated for the 64 included studies, with each data point representing an individual study. The funnel plots for ALB, SCr, TTPR, TTHR, and AE incidence showed an approximately symmetrical distribution of data points around the midline, suggesting a low likelihood of publication bias in these outcomes. In contrast, the funnel plot for 24hU-Pro exhibited asymmetry, indicating potential publication bias or small-study effects, as shown in **Figure 7**. The observed bias may stem from the fact that many of the included studies were single-center investigations with small sample sizes (n < 100), which reduces statistical power and may lead to under-reporting of negative results, thereby contributing to publication bias.

Furthermore, the asymmetric point distribution in the plot, with a concentration in the positive effect size region (right side), warrants further consideration. While studies with larger standard errors (upper part of the plot) are distributed on both sides, those with small to moderate errors (middle to lower part) are distinctly skewed to the right. Two additional factors may explain this pattern. First, studies demonstrating a significant reduction in proteinuria with immunosuppressive agents may be more likely to be published, while those with negative or null findings might remain unpublished. Second, clinical experience suggests that achieving complete or partial remission of significant proteinuria often requires several months (6–12 months) of continuous immunosuppressive therapy. Some of the included RCTs had treatment durations of only 6–8 weeks; thus, the “endpoint proteinuria” they measured likely reflects an “initial treatment response” rather than the “final remission rate.” This would lead to an underestimation of the true efficacy of the interventions, introduce substantial heterogeneity, and—if treatment durations varied widely across studies (e.g., from 4 weeks to 12 months)—result in measurements capturing effects at different treatment stages. This variability yields unreliable results and is visualized as scattered points in the funnel plot. Notably, the clear rightward skew in the funnel plot for adverse events may suggest that receiving immunosuppressive interventions could be associated with an increased risk of adverse reactions.

## 5 Discussion

### 5.1 Summary of main results

This systematic review and network meta-analysis represents the first comprehensive evaluation of the efficacy and safety of nine distinct immunosuppressive regimens for the treatment of Henoch-Schönlein purpura nephritis (HSPN) in pediatric patients. The analysis incorporates data from 64 randomized controlled trials (RCTs) encompassing a total of 4,931 participants. Utilizing Surface Under the Cumulative Ranking (SUCRA) metrics and cluster analysis, the study identifies specific advantages of different regimens for targeted clinical outcomes. The combination of mycophenolate mofetil with conventional therapy (MMF+CT) was found to be most effective in reducing 24-hour urine protein (24hU-Pro), increasing serum albumin (ALB), and decreasing blood urea nitrogen levels. Tacrolimus combined with conventional therapy (TAC+CT) was superior in enhancing serum creatinine (SCr) levels. In terms of expediting the resolution of clinical symptoms, Tripterygium wilfordii polyglycoside with conventional therapy (TWP+CT) significantly accelerated the resolution of hematuria and proteinuria. Regarding safety profiles, cyclosporine A combined with conventional therapy (CsA+CT) was associated with the lowest incidence of adverse events, while mycophenolate mofetil combined with conventional therapy (MMF+CT) offered the most favorable balance between efficacy and safety.

### 5.2 Comparison with other studies

Numerous systematic reviews and meta-analyses have substantiated the substantial clinical efficacy of immunosuppressive agents, such as cyclophosphamide and tacrolimus, in the management of pediatric Henoch-Schönlein purpura nephritis (HSPN), particularly in terms of ameliorating proteinuria and preserving renal function (78, 79). Evidence suggests that long-term combination therapy involving glucocorticoids and immunosuppressants is more effective in controlling inflammatory responses; however, extended use may result in diminished renal function and other adverse effects (80). Consequently, vigilant monitoring of renal function and potential side effects is imperative during prolonged treatment to ensure both safety and efficacy. These findings corroborate the general conclusion regarding the effectiveness of immunosuppressive interventions presented in our study. It is important to note that the Kidney Disease: Improving Global Outcomes (KDIGO) guidelines (81) address IgA vasculitis (IgAV) without age restriction and do not endorse any specific immunosuppressive combination regimen for IgAV, merely indicating that the drugs employed for IgA nephropathy (IgAN) have not been validated for use in IgAV. Moreover, the KDIGO guidelines provide limited evidence regarding IgA vasculitis (IgAV), lack specific efficacy targets, primarily focus on minimizing unnecessary steroid use, and do not address indicators such as serum albumin levels or the time required for the resolution of hematuria/proteinuria.

### 5.3 Explanation of the research results

Our results show that MMF+CT demonstrated the best effect in reducing 24hU-Pro and elevating ALB, with SUCRA ranking confirming it as the optimal intervention for these outcomes. This finding has a pathophysiological basis. The pathogenesis of HSPN closely resembles that of IgA nephropathy (IgAN), centered on the deposition of galactose-deficient IgA1 (Gd-IgA1) immune complexes in the mesangium, which activates the complement system and triggers an inflammatory cascade (82, 83). MMF, as a selective inhibitor of inosine monophosphate dehydrogenase (IMPDH), specifically blocks the *de novo*synthesis pathway of guanine nucleotides. Since T and B lymphocytes are highly dependent on this pathway for proliferation, MMF effectively suppresses antibody production by B cells, thereby reducing the formation of pathogenic IgA1 immune complexes and their deposition in the glomeruli at the source. Furthermore, studies indicate that MMF can also inhibit the excessive proliferation of mesangial cells and protect vascular endothelial cells and podocytes by reducing oxidative stress (84), thereby helping to maintain the integrity of the glomerular filtration barrier. This explains its superior performance in reducing proteinuria (24hU-Pro) and improving hypoalbuminemia (ALB).

Tacrolimus (TAC) combined with conventional therapy performed best in lowering serum creatinine (SCr) levels (SUCRA = 97.0%). This advantage may be attributed not only to its classic immunomodulatory effects—namely, inhibiting T-cell activation and IL-2 production (85)—but also to its unique non-immunologic protective mechanisms.

First, TAC can directly stabilize the podocyte cytoskeleton, thereby repairing the glomerular filtration barrier. As a calcineurin inhibitor (CNI), TAC not only stabilizes the cytoskeleton by regulating WAVE1 phosphorylation (85) but also ameliorates podocyte dysfunction by restoring the expression of FK506 binding protein 12 (FKBP12) in podocytes (86). The integrity of the podocyte cytoskeleton is crucial for maintaining renal function: studies show that deletion of the key protein Talin1 leads to severe proteinuria and renal failure (87), while the podocyte-specific protein Rhophilin-1 maintains cellular integrity by regulating actin organization (88). By intervening in these mechanisms, TAC exerts significant cytoprotective effects.

Second, regarding hemodynamics, TAC may improve renal function by modulating intraglomerular pressure. Research suggests that TAC may reduce glomerular hypertension by affecting afferent arteriolar tone through a mechanism similar to endothelin receptor antagonists (ERAs), an effect that can contribute to a significant short-term reduction in creatinine levels (89). Furthermore, activation of the calcium-sensing receptor can stabilize the podocyte cytoskeleton and improve cell survival, indicating that TAC might further mitigate toxin-induced glomerulosclerosis by modulating calcium signaling pathways (85).

However, the vascular effects of TAC are double-edged. Specifically, it may increase vasoconstriction via the RhoA/ROCK pathway. While this effect can help reduce glomerular hyperfiltration, it may lead to nephrotoxicity and other complications in the long term (90). Some studies suggest this side effect might be mitigated by co-administration with calcium channel blockers (91).

Despite potential risks, TAC remains a priority consideration for children with HSPN accompanied by renal insufficiency. Existing evidence shows that TAC achieves high remission rates in treating steroid-resistant nephrotic syndrome in children and carries a relatively lower risk of acute renal function deterioration compared to other immunosuppressants (79, 92). Furthermore, when used in combination with a low-dose strategy, TAC has been shown to significantly reduce the incidence of chronic kidney disease (93, 94), which is particularly important for children requiring long-term maintenance therapy.

In summary, although calcineurin inhibitors demonstrate significant non-immunologic protective advantages in HSPN treatment, their potential nephrotoxicity warrants caution. Clinical decisions should be individualized based on the patient’s specific condition and risk assessment to maximize efficacy while minimizing adverse effects (90, 95).

Regarding the speed of clinical symptom resolution, our study indicates that Tripterygium wilfordii polyglycoside combined with conventional therapy (TWP+CT) can significantly shorten the time to resolution of hematuria and proteinuria. This rapid onset of action is primarily attributed to its potent multi-target anti-inflammatory mechanisms. Studies have confirmed that TWP effectively mitigates glomerular microinflammation by inhibiting key signaling pathways such as p38 MAPK and NF-κB (96). Concurrently, it suppresses the pathological proliferation of mesangial cells by promoting autophagy through modulation of the CARD9/p38 MAPK pathway (97). Furthermore, TWP exhibits significant synergistic effects with other drugs (e.g., RAS system inhibitors), with clinical evidence showing that combination therapy effectively improves remission rates and further reduces proteinuria excretion (98). However, given the potential toxic risks of TWP, particularly hepatotoxicity associated with long-term use (99), a careful risk-benefit assessment is mandatory in clinical application. Strategies to mitigate adverse reaction risks are recommended, including rigorous safety monitoring, dose adjustment, or combination therapy approaches (100).

In terms of safety, our results indicate that cyclosporine A combined with conventional therapy (CsA+CT) was associated with the relatively lowest risk of adverse events. All interventions demonstrated acceptable safety profiles, with serious adverse events being rare. However, it is noteworthy that the nephrotoxicity of CsA remains a primary clinical concern, mainly manifesting as renal interstitial fibrosis, gingival hyperplasia, and hypertrichosis (101). These effects not only impact patients’ quality of life but may also lead to long-term renal impairment.

Renal interstitial fibrosis induced by CsA is a key manifestation of its nephrotoxicity. Studies show that CsA promotes renal interstitial fibrosis by upregulating signaling pathways such as Nox2 and TGF-β1 (102). Furthermore, by inhibiting calcineurin activity, CsA leads to apoptosis and dysregulated proliferation of renal tubular epithelial cells, thereby exacerbating interstitial fibrosis (103). This fibrosis not only affects renal structural integrity but can also result in progressive decline in renal function (104).

The discrepancy between our findings and clinical perception regarding CsA’s safety profile may be explained by two factors. First, most included RCTs had short follow-up periods (less than 6 months). CsA’s nephrotoxicity may not be significant in the short term, making it a viable option for certain acute treatments (105). In contrast, gastrointestinal reactions from CTX and infections associated with MMF are acute and more readily documented in short-term studies. Second, CsA may mitigate its own nephrotoxicity under certain conditions by modulating lipid metabolism and reducing oxidative stress (106).

Therefore, when using CsA for immunosuppressive therapy, its potential nephrotoxicity risk must be comprehensively considered, and corresponding preventive measures should be implemented, such as dose adjustment or combination with other immunosuppressants (107).

In the pediatric population, safety is a paramount consideration. Our cluster analysis visually integrated efficacy and safety, identifying MMF+CT as the best-performing regimen overall. Conversely, although CTX+CT remains a standard therapy, our analysis categorized it into the cluster characterized by “fast symptom resolution but low safety.” The adverse event rate in the CTX+CT group was notable, and its safety SUCRA score consistently ranked low. While CsA+CT ranked highest in safety (SUCRA = 85.2%), these findings challenge the traditional reliance on CTX for all severe cases and advocate for a shift towards less toxic agents like MMF, especially considering pediatric patients’ susceptibility to long-term side effects such as gonadotoxicity.

In the loop inconsistency test for serum creatinine (SCr), significant statistical inconsistency was observed in the closed loop formed by CTX+CT, TAC+CT, and MMF+CT [IF = 22.16, 95% CI (11.74, 32.58), P = 0.00]. This result indicates a discrepancy between direct and indirect comparisons, and the large absolute value of IF further suggests a potential tendency for inconsistency, which may be related to various inherent sources of heterogeneity in the network meta-analysis. First, clinical heterogeneity within the conventional therapy (CT) groups is a key factor contributing to this inconsistency. Due to the lack of unified clinical practice guidelines for pediatric HSPN (IgA vasculitis nephritis), the specific composition and implementation details of CT varied significantly across studies. For instance, some studies used different doses and durations of glucocorticoids, while others combined supportive therapies such as antihypertensives, antiproteinuric agents, or dietary interventions. Such variations in the core components of CT may lead to differences in baseline therapeutic effects across study populations, thereby interfering with the relative efficacy comparison between immunosuppressants and ultimately causing inconsistency between direct and indirect comparisons. Second, differences in baseline renal function among included patients likely exacerbated this inconsistency. Inevitable variations existed across studies in patients’ SCr levels, estimated glomerular filtration rate (eGFR), and severity of proteinuria. Patients with impaired baseline renal function, whose renal pathological damage and regenerative capacity differ from those with normal function, may respond differently to combination therapy with immunosuppressants (CTX, TAC, MMF) and CT. Furthermore, other potential factors cannot be excluded. For example, heterogeneity in intervention protocols (e.g., differences in the dose, route of administration, and treatment duration of CTX, TAC, or MMF across studies) may influence the effect on SCr. Variations in follow-up duration may lead to different time points for SCr measurement, as improvements or deteriorations in renal function may follow different trends at different treatment stages. Additionally, methodological heterogeneity among the included studies (e.g., differences in sample size calculation, blinding design, and handling of missing data) could introduce bias into the results, affecting the consistency between direct and indirect comparisons.

Future research should focus on standardizing the definition and implementation of CT, rigorously controlling for confounding factors like baseline renal function, and employing uniform intervention protocols and methodological standards to reduce heterogeneity, thereby enhancing the reliability and validity of network meta-analysis findings.

### 5.4 Limitations and strengths

This study, encompassing 64 randomized controlled trials (RCTs), represents the inaugural application of network meta-analysis (NMA) within this specific context, facilitating indirect comparisons of treatments not previously evaluated in direct head-to-head trials. The incorporation of cluster analysis further enables clinicians to visually examine the trade-offs between efficacy and safety.

Several limitations of this study merit careful consideration. Firstly, the predominance of studies published in Chinese (63 out of 64) introduces a significant geographical and ethnic bias, rendering our findings directly applicable only to Chinese pediatric populations. This limitation is particularly salient given that the 2021 International Pediatric Nephrology Association (IPNA) (108) guidelines on the treatment of IgA nephropathy and IgA vasculitis nephritis recommend against the use of mycophenolate mofetil (MMF) except in Chinese and Korean patients, where data suggest potential benefits. Consequently, our finding that MMF+CT offers the best efficacy-safety balance must be interpreted within this specific ethnic context and should not be extrapolated to other populations without dedicated validation. Moreover, although pediatric and adult IgAV share the core pathogenesis of galactose-deficient IgA1 (Gd-IgA1) immune complex deposition and complement activation (e.g., C3a/C5a), their clinical courses differ markedly. As reviewed by González-Gay et al. (109) and Castañeda et al. (110), adult IgAV is rare but substantially more severe: 40–85% of adults with IgAV develop nephritis, and 10-30% progress to end-stage renal disease, whereas pediatric IgAV is more common but usually self-limiting with milder renal involvement. These differences indicate that our pediatric findings should not be directly extrapolated to adult patients without dedicated validation. Secondly, the near-absence of blinding represents a major methodological concern. As shown in **Figure 2**, 63 out of 64 included studies (98.4%) lacked blinding, resulting in an “unclear risk” classification for performance and detection bias according to the Cochrane Risk of Bias tool. This open-label design may introduce bias in outcome assessment, particularly for subjective endpoints, and may have influenced both physicians’ and patients’ expectations regarding treatment response and adverse event reporting. Readers should interpret the findings with this important limitation in mind. Notably, regarding the six studies rated as having incomplete outcome data (“some concerns”), it should be clarified that these studies were included only in the qualitative synthesis (study characteristics description and risk of bias assessment) because they did not report complete outcome data, and they were not incorporated into the quantitative NMA. Thirdly, the visual asymmetry detected in the funnel plot for 24-hour urinary protein (24hU-Pro) suggests the presence of publication bias or “small-study effects,” wherein studies with negative outcomes or smaller sample sizes may remain unpublished. Fourthly, there was considerable heterogeneity in the treatment duration among the included RCTs. As highlighted in our analysis, numerous studies featured short follow-up periods (6–8 weeks), whereas achieving complete remission in Henoch-Schönlein purpura nephritis (HSPN) often necessitates several months. Consequently, the reported “endpoint” proteinuria levels may primarily indicate an early treatment response rather than the long-term remission rate, potentially underrepresenting the true efficacy of agents with a slower onset of action. Finally, it should be noted that none of the included RCTs reported glomerular filtration rate (GFR) as an outcome measure; all studies uniformly used serum creatinine (SCr) as the primary indicator of renal function. Given the significant variability in body size, age, and muscle mass among pediatric patients, SCr alone may not accurately reflect true renal function in children. GFR, whether estimated or measured, would be a more appropriate and standardized endpoint for future trials to enable more reliable cross-study comparisons. We strongly recommend that subsequent studies in pediatric HSPN adopt GFR as a core renal function outcome. Furthermore, due to the lack of detailed data from the original studies, we were unable to conduct comprehensive subgroup analyses or meta-regression based on pathological classifications (e.g., ISKDC grades) or clinical subtypes of Henoch-Schönlein purpura nephritis (HSPN). This limitation hinders our ability to provide more tailored treatment recommendations for children with varying levels of disease severity.

## 6 Conclusion

In conclusion, this network meta-analysis demonstrates that, compared to conventional therapy alone, immunosuppressive agents significantly enhance treatment outcomes in children with HSPN. The combination of mycophenolate mofetil with conventional therapy (MMF+CT) achieves an optimal balance between efficacy and safety, particularly in managing proteinuria and hypoalbuminemia. In contrast, tacrolimus combined with conventional therapy (TAC+CT) is more appropriate for patients with compromised renal function, while Tripterygium wilfordii polyglycoside combined with conventional therapy (TWP+CT) demonstrates effectiveness in rapidly controlling symptoms. These findings advocate for a transition towards individualized, phenotype-driven treatment strategies. Future research should prioritize the execution of large-scale, multicenter, long-term randomized controlled trials (RCTs) to validate the sustainability of these outcomes and to establish standardized criteria for reporting adverse events.

## Data Availability

The original contributions presented in the study are included in the article/**Supplementary Material**. Further inquiries can be directed to the corresponding author.
