# Supplementary material for: Efficacy and safety of various immunosuppressive agents for pediatric Henoch-Schönlein purpura nephritis (IgA vasculitis nephritis) in Chinese children: a systematic review and network meta-analysis
Source: Front Immunol. 2026 Jul 31;17:1868631. doi: 10.3389/fimmu.2026.1868631 (PMC13473287; doi:10.3389/fimmu.2026.1868631)
Supplement: Supplementary file 1 [file Table1.docx]

**SUPPLEMENTARY MATERIAL**

**Table of Contents**

**Supplementary Methods 1 –** Database search strategy.

**Supplementary Methods** **2 –** Conversion formula for standard deviation (SD).

**Supplementary Table S1 –** Explanation of technical terms.

**Supplementary Table S****2 –** Demographic characteristics of included studies

**Supplementary Table S3 –** Results of network Meta-Analysis (24hU-Pro).

**Supplementary Table S4 –** Results of network Meta-Analysis(ALB).

**Supplementary Table S5 –** Results of network Meta-Analysis(SCr).

**Supplementary Table S6 –** Results of network Meta-Analysis(BUN).

**Supplementary Table S7 –** Results of network Meta-Analysis(TTPR).

**Supplementary Table S8 –** Results of network Meta-Analysis(TTHR).

**Supplementary Table S9 –** Results of network Meta-Analysis(AE incidence).

**Supplementary Table S10 –**Treatment rankings and SUCRA values from the network meta-analysis for each outcome.

**Supplementary Figure S1 –** Risk of bias assessment included in the study.

**Supplementary Methods 1 –** Database search strategy.

**Pubmed**

((("Glomerulonephritis, IGA"[Mesh]) OR (((((((((((((Glomerulonephritides, IGA[Title/Abstract]) OR (Berger's Disease[Title/Abstract])) OR (Bergers Disease[Title/Abstract])) OR (IGA Glomerulonephritis[Title/Abstract])) OR (Nephropathy, IGA[Title/Abstract])) OR (Iga Nephropathy 1[Title/Abstract])) OR (Nephropathy 1, Iga[Title/Abstract])) OR (Immunoglobulin A Nephropathy[Title/Abstract])) OR (Nephropathy, Immunoglobulin A[Title/Abstract])) OR (Nephritis, IGA Type[Title/Abstract])) OR (IGA Type Nephritis[Title/Abstract])) OR (Berger Disease[Title/Abstract])) OR (IGA Nephropathy[Title/Abstract]))) OR ((("IgA Vasculitis"[Mesh]OR "Purpura, Schoenlein-Henoch"[Title/Abstract]OR "Henoch-Schönlein purpura"[Title/Abstract] OR "HSP"[Title/Abstract] OR "anaphylactoid purpura"[Title/Abstract])) AND (("nephritis"[Title/Abstract] OR "renal"[Title/Abstract] OR "kidney"[Title/Abstract])))) AND ((("Immunosuppressive Agents" OR "Agents, Immunosuppressive" OR "Immunosuppressants" OR "Immunosuppressive Agent" OR "Agent, Immunosuppressive" OR "Immunosuppressant" OR "Immunosuppressive drugs" OR "Cyclophosphamide" OR "Cyclosporine" OR "Cyclosporine A" OR "Tacrolimus" OR "Mycophenolate mofetil" OR "MMF" OR "Rituximab" OR "Cyclosporin" OR "Azathioprine" OR "Mizoribine" OR "Vincristine" OR "Calcineurin inhibitors" OR "Mycophenolic sodium" OR "MPS" )))

**Web of Science**

#1

TS=(Glomerulonephritis, IGA) OR (Glomerulonephritides, IGA) OR (Berger's Disease) OR (Bergers Disease) OR (IGA Glomerulonephritis) OR (Nephropathy, IGA) OR (Iga Nephropathy 1) OR (Nephropathy 1,iga) OR (Immunoglobulin A Nephropathy) OR (Nephropathy, Immunoglobulin A) OR (Nephritis, IGA Type) OR (IGA Type Nephritis) OR (Berger Disease) OR (IGA Nephropathy)

#2

TS=((IgA Vasculitis) OR (Henoch-Schönlein purpura) OR (HSP) OR (anaphylactoid purpura)) AND ((nephritis) OR (renal) OR (kidney))

#3

TS=(Immunosuppressive Agents) OR (Agents, Immunosuppressive) OR (Immunosuppressants) OR (Immunosuppressive Agent) OR (Agent, Immunosuppressive) OR (Immunosuppressant) OR (Immunosuppressive drugs) OR (Cyclophosphamide) OR (Cyclosporine) OR (Cyclosporine A) OR (Tacrolimus) OR (Mycophenolate mofetil) OR (MMF) OR (Rituximab) OR (Cyclosporin) OR (Azathioprine) OR (Mizoribine) OR (Vincristine) OR (Calcineurin inhibitors) OR (Mycophenolic sodium) OR (MPS)

(#1 OR #2)AND #3

**Cochrane Library**

#1 MeSH descriptor: [Glomerulonephritis, IGA] explode all trees

#2 (Glomerulonephritides, IGA OR Berger's Disease OR Bergers Disease OR IGA Glomerulonephritis OR Nephropathy, IGA OR IgA Nephropathy 1 OR Nephropathy 1, Iga OR Immunoglobulin A Nephropathy OR Nephropathy, Immunoglobulin A OR Nephritis, IGA Type OR IGA Type Nephritis OR Berger Disease OR IGA Nephropathy):ti,ab,kw

#3 #1 OR #2

#4 MeSH descriptor: [IgA Vasculitis] explode all trees

#5 (Purpura, Schoenlein-Henoch OR Henoch-Schönlein purpura OR HSP OR anaphylactoid purpura):ti,ab,kw

#6 (nephritis OR renal OR kidney):ti,ab,kw

#7 #5 AND #6

#8 #4 OR #7

#9 #3 OR #8

#10 (Immunosuppressive Agents OR Agents, Immunosuppressive OR Immunosuppressants OR Agent, Immunosuppressive):ti,ab,kw

#11 (Immunosuppressant OR Immunosuppressive drugs OR Cyclophosphamide OR Cyclosporine):ti,ab,kw

#12 (Tacrolimus OR Mycophenolate mofetil OR MMF OR Rituximab OR Cyclosporin):ti,ab,kw

#13 (Azathioprine OR Mizoribine OR Vincristine OR Calcineurin inhibitors OR Mycophenolic sodium):ti,ab,kw

#14 (MPS):ti,ab,kw

#15 #10 OR #11 OR #12 OR #13 OR #14

#16 #9 AND #15

**Embase**

#1 'immunoglobulin a nephropathy'/exp

#2 (glomerulonephritides, iga OR bergers disease OR iga glomerulonephritis OR nephropathy, iga OR iga nephropathy 1 OR nephropathy 1, iga OR immunoglobulin a nephropathy OR nephropathy, immunoglobulin a OR nephritis, iga type OR iga type nephritis OR iga nephropathy):ab,ti

#3 #1 OR #2

#4 'anaphylactoid purpura'/exp

#5 (iga vasculitis OR purpura, schoenlein-henoch OR henoch-schonlein purpura OR hsp OR anaphylactoid purpura):ab,ti

#6 (nephritis OR renal OR kidney):ab,ti

#7 #4 OR #5

#8 #6 AND #7

#9 #3 OR #8

#10 (immunosuppressive agent* OR agents, immunosuppressive OR immunosuppressants OR agent, immunosuppressive OR immunosuppressant OR immunosuppressive drug* OR cyclophosphamide OR cyclosporine OR tacrolimus OR mycophenolate mofetil OR mmf OR rituximab OR cyclosporin OR azathioprine OR mizoribine OR vincristine OR calcineurin inhibitor* OR mycophenolic sodium OR mps):ab,ti

#11 #9 AND #10

#12 #11 AND (clinical article/de OR 'controlled study'/de)

**Supplementary Methods 2 –** Conversion formula for standard deviation (SD).

**Standard Error to Standard Deviation:**

SD=SE×$\sqrt{N}$ where SE is the standard error and N is the sample size.

**95% Confidence Interval to Standard Deviation:**

1. For sample sizes in the test and control groups that are greater than or equal to 100, the standard deviation (SD) can be calculated as follows: SD = (Upper limit of credible intervals - Lower limit of credible intervals) / 3.92.
2. For sample sizes in the test and control groups that are less than or equal to 60, the standard deviation (SD) is determined by the formula: SD = (Upper limit of credible intervals - Lower limit of credible intervals) / tinv(1-0.95, n-1). 3. For studies where the sample sizes in each group range between 60 and 100, either of the aforementioned methods may be employed.

**Combined Subgroup**

In the combined subgroup analysis, let the sample size of subgroup A be denoted as N_1_, with a mean of M_1_ and a standard deviation of SD_1_. For subgroup B, let the sample size be N_2_, with a mean of M_2_ and a standard deviation of SD_2_. The total combined sample size is (N = N_1_ + N_2_). The combined mean M is calculated as (M = N_1_M_1_+ N_2_M_2_)/(N_1_ + N_2_).

**Supplementary Table S1 Explanation of technical terms.**

| **Standardized mean difference (SMD)** | The SMD serves as an effect size metric employed to quantify the mean difference between two data groups. By normalizing the mean difference between groups through division by the pooled standard deviation, it mitigates the influence of dimensionality, thereby facilitating the comparability of effect sizes across diverse studies or variables. This standardization enables the translation of differences into a common metric, allowing for the comparison of studies conducted on varying scales or units. |
| --- | --- |
| **95% credible intervals (CI)** | In the context of frequentism metwork meta-analysis, the 95% confidence interval (CI) serves as a fundamental tool for evaluating the uncertainty associated with the effects of interventions. This approach is grounded in classical statistical theory, which posits that the effect size is a fixed parameter. The CI quantifies the "true effect" by synthesizing both direct and indirect comparison evidence, thereby delineating the potential range of effect sizes. Its application spans several critical scenarios: it can quantify the impact of intervention measures (such as risk ratios and mean differences) and determine statistical significance by assessing whether the CI includes null values (for instance, whether the CI encompasses 0 for continuous variables or 1 for binary variables). Furthermore, the CI can assess the reliability of evidence by examining the extent of overlap between direct and indirect evidence in consistency tests. Unlike the credible interval used in Bayesian network meta-analysis, the 95% CI in this context is fundamentally an inference about true values under repeated sampling, emphasizing objective evidence inference. It provides a rigorous statistical framework for integrating complex evidence networks and supports evidence-based decision-making. |
| **Statistical difference** | In the context of frequentism metwork meta-analysis, the determination of statistical significance is grounded in classical statistical theory. The primary objective is to assess whether the observed differences between interventions are "non-random" by examining the effect size and its corresponding 95% confidence interval (CI). For relative effect measures, such as the hazard ratio (HR) or odds ratio (OR), a 95% CI that does not encompass the null value of 1 indicates a statistically significant difference. Similarly, for absolute effect measures, such as the mean difference (MD) or standardized mean difference (SMD), a 95% CI that does not include 0 signifies statistical significance. This evaluative criterion is applied consistently, regardless of whether the comparison is a direct head-to-head test or an indirect comparison facilitated through an intermediate intervention. |
| **Heterogeneity** | In the context of meta-analysis, heterogeneity pertains to the variability among included studies, which can manifest across various dimensions such as study outcomes, methodologies, population characteristics, or intervention strategies. This variability fundamentally indicates that the effect size of each individual study cannot be adequately accounted for by a single common effect. The concept of heterogeneity encompasses statistical, clinical, and methodological dimensions. Statistical heterogeneity is assessed by quantifying the degree of variation in effect sizes, with the I² statistic frequently employed as a measure; for instance, an I² value exceeding 50% is indicative of moderate to high heterogeneity. |
| **SUCRA (Surface Under the Cumulative RANK Curve)** | In the context of network meta-analysis, SUCRA (Surface Under the Cumulative RANK Curve) serves as a metric to assess the likelihood of ranking the relative efficacy of various interventions. This measure effectively consolidates the ranking probabilities of different interventions into a single value ranging from 0 to 1, facilitating intuitive comparison and ranking. A SUCRA value approaching 1 suggests that an intervention is more likely to be effective, whereas a value nearing 0 indicates a higher likelihood of being the least effective treatment. By weighting the cumulative probabilities of all potential ranking positions, SUCRA synthesizes evidence from both direct and indirect comparisons, thereby overcoming the limitations associated with rankings based solely on point estimates. Additionally, the 95% confidence interval provides insight into the stability of these rankings. As a non-parametric measure, SUCRA does not require assumptions regarding the distribution of effect sizes. In the context of network comparisons involving multiple interventions, SUCRA offers a quantitative basis for clinical decision-making, aiding researchers and policymakers in identifying the most favorable interventions. |

**Supplementary Table S2 Demographic characteristics of included studies.**

| Paper | Diagnostic criteria | Sample size (Control group/  Treatment group/  Other groups) | Gender  (M/F) | Control group | Treatment group | Other group | Intervention  Control group | InterventionTreatment group | InterventionOther group | Age  (years)  Control group | Age  (years)  Treatment group | Age  (years)Other group | Duration of treatment  (month） | Outcomes | randomization method |
| --- | --- | --- | --- | --- | --- | --- | --- | --- | --- | --- | --- | --- | --- | --- | --- |
| Ma2019 | 24hUpro > 150mg | 150/150 | 155/145 | 77/73 | 78/72 | — | CTX8-12mg/(kg·d)+PRED2mg/(kg·d)+DIPY | MMF20-30mg/(kg·d)+PRED2mg/(kg·d)+DIPY | — | 8.45 ± 2.23 | 8.65 ±2.16 | — | 6 | ①③ | Random |
| Liu2017 | CMA2000 | 45/45 | 51/39 | 26/19 | 25/20 | — | PRED1.5-2mg/(kg·d)+VC+DIPY | TWP1mg/(kg·d)+PRED0.5-1mg/(kg·d)+VC+DIPY | — | 6.31±2.05 | 6.81±2.14 | — | unclear | ②⑥⑦ | Random Number Table |
| Zhang2017 | NA | 35/35 | 39/31 | 19/16 | 20/15 | — | PRED1.5-2mg/(kg·d) | TWP1mg/(kg·d)+PRED1.5-2mg/(kg·d) | — | 7.08±1.54 | 4.52±0.97 | — | 2 | ① | Random |
| Guo2020 | CMA2013 | 36/36 | 42/30 | 22/14 | 20/16 | — | TAC 0.05-0.1mg/(kg·d)+PRED 10mg/d | MMF 0.75-1g/d+PRED 10mg/d | — | 6.52±3.74 | 6.81±3.35 | — | 3 | ①③ | Random Number Table |
| Wu2020 | NA | 43/43 | 45/41 | 22/21 | 23/20 | — | CTX 8-12mg/(kg·d)+PRED2mg/(kg·d) | TAC 0.1-0.15mg/(kg·d)+PRED2mg/(kg·d) | — | 6.4±2.5 | 6.8±2.7 | — | 6 | ② | Random |
| Yang2018 | NA | 43/43 | 54/32 | 28/15 | 26/17 | — | CTX 9mg/(kg·d)+PRED10-20mg/(kg·d) | MMF 20mg/(kg·d)+PRED1mg/(kg·d) | — | 8.3±0.19 | 6.8±2.3 | — | 6 | ①③ | Random Number Table |
| Wang2012 | CMA2003 | 30/30 | 39/21 | 19/11 | 20/10 | — | CTX 10mg/(kg·d)+MP15-20mg/(kg·d)+DIPY5-10mg/(kg·d)+UFH0.5-1mg/(kg·d)+VC40-80mg/(kg·d)+TROXE4-8mg/(kg·d)+CPA0.35mg/(kg·d)+CIM12-16mg/(kg·d) | MMF 20mg/(kg·d)+MP15-20mg/(kg·d)+DIPY5-10mg/(kg·d)+UFH0.5-1mg/(kg·d)+VC40-80mg/(kg·d)+TROXE4-8mg/(kg·d)+CPA0.35mg/(kg·d)+CIM12-16mg/(kg·d) | — | 10.5±4.5 | 10.0±5.0 | — | 12 | ①②③④ | Random |
| Fang2020 | NA | 52/52 | 58/46 | 28/24 | 30/22 | — | CTX 10mg/(kg·d)+PRED0.5-1mg/(kg·d) | MMF 15-30mg/(kg·d)+PRED0.5-1mg/(kg·d) | — | 7.50±2.80 | 7.80±2.20 | — | 6 | ⑥⑦ | Random |
| Wang2019 | CMA2009 | 46/46 | 56/36 | 29/17 | 27/19 | — | PRED30mg/d+VC600mg/d+TROXE240mg/d+CaD3 600mg/d | TWP1.5mg/(kg·d)+VC600mg/d+TROXE240mg/d+CaD3 600mg/d | — | 5.4±2.5 | 6.4±2.5 | — | 1 | ⑥⑦ | Random |
| Li2014 | CMA2009 | 35/32 | 40/27 | 21/14 | 19/13 | — | PRED 1mg+C.R.120mg/d+VE 200mg/d+DIPY3mg/(kg·d) | TWP1.5mg/(kg·d)+PRED 1mg+C.R.120mg/d+VE 200mg/d+DIPY3mg/(kg·d) | — | 7.1±3.6 | 6.8±3.3 | — | 3 | ② | Random Number Table |
| Gu2016 | NA | 44/44 | 48/40 | 23/21 | 25/19 | — | PRED2mg/(kg·d)+DIPY3mg/(kg·d) | TWP1-1.5mg/(kg·d)+PRED2mg/(kg·d)+DIPY3mg/(kg·d) | — | 7.2±2.2 | 7.4±2.1 | — | 3 | ② | Random |
| Yan2013 | NA | 45/45 | 49/41 | 25/20 | 24/21 | — | PRED1.5-2mg/(kg·d)+DIPY+VC+Ca | TWP1mg/(kg·d)+PRED1.5-2mg/(kg·d)+DIPY+VC+Ca | — | 4.51±0.83 | 4.23±0.65 | — | unclear | ②⑥⑦ | Random |
| Xiang2014 | CMA2009 | 21/21 | 22/20 | 10/11 | 12/9 | — | PRED+DIPY+VC+Ca | TWP1mg/(kg·d)+PRED+DIPY+VC+Ca | — | 6.82±3.36 | 6.36±3.21 | — | 3 | ②⑥⑦ | Random Number Table |
| Niu2019 | NA | 46/46 | 55/37 | 27/19 | 28/18 | — | MP15-20mg/(kg·d) | TWP1mg/(kg·d)+MP 15-20mg/(kg·d) | — | 8.73±1.96 | 8.42±2.05 | — | 2 | ② | Random Number Table |
| Yang2016 | CMA2001 | 42/42 | 50/34 | 24/18 | 26/16 | — | MP1mg/(kg·d)+DIPY3mg/(kg·d) | TWP1.5mg/(kg·d)+MP1mg/(kg·d)+DIPY3mg/(kg·d) | — | 8.6±2.4 | 8.3±2.6 | — | 3 | ① | Random Number Table |
| Dai2006 | 24hUpro > 3.5g/24h | 23/25 | 32/16 | 15/8 | 17/8 | — | PRED1.5-2mg/(kg·d)+DIPY1-2g/(kg·d) | CTX8-10mg/(kg·d)+PRED1.5-2mg/(kg·d)+DIPY1-2g/(kg·d) | — | 11.3±3.2 | 10.5±4.2 | — | 4 | ①③⑤ | Random |
| Liu2019 | NA | 20/20 | 21/19 | 9/11 | 12/8 | — | CTX10mg/(kg·d)+PRED1.5-2mg/(kg·d) | MMF15-20mg/(kg·d)+PRED1.5-2mg/(kg·d) | — | 9±1.5 | 8.2±2.2 | — | unclear | ⑥⑦ | Random |
| Lv2019 | NA | 52/52 | 58/46 | 30/22 | 28/24 | — | CTX10mg/(kg·d)+PRED0.5-1mg/(kg·d) | MMF 15-30mg/(kg·d)+PRED0.5-1mg/(kg·d) | — | 7.8±2.2 | 7.5±2.8 | — | 6 | ②⑥⑦ | Random Number Table |
| Wang2012-2 | CMA2001CMA2009 | 54/50 | 56/48 | 26/28 | 30/20 | — | PRED1.5-2mg/(kg·d)+DIPY+VC+Ca | TWP1mg/(kg·d)+PRED 1.5-2mg/(kg·d)+DIPY+VC+Ca | — | 6. 82±3.36 | 6. 36±3.21 | — | 3-6 | ⑥⑦ | Random Number Table |
| Huo2011 | CMA2000 | 30/30 | 33/27 | 16/14 | 17/13 | — | PRED30mg/d | LEF0.8mg/(kg·d)+PRED 20mg/d | — | unclear | unclear | — | 3 | ①③④⑤ | Random |
| Huang2015 | CMA2009 | 30/30 | 29/31 | unclear | unclear | — | PRED1.5-2mg/(kg·d)+DIPY3-5mg/(kg·d)+VC+Ca | MMF20-30mg/d+PRED1.5-2mg/(kg·d)+DIPY3-5mg/(kg·d)+VC+Ca | — | unclear | unclear | — | unclear | ② | Random |
| Gan2024 | NA | 30/30 | 37/23 | 18/12 | 19/11 | — | CIM10-15mg/(kg·d)+VC0.1-0.3g/d+LOR5-10mg/d+DEX0.25mg/(kg·d) | TAC0.1mg/(kg·d)+CIM10-15mg/(kg·d)+VC0.1-0.3g/d+LOR5-10mg/d+DEX0.25mg/(kg·d) | — | 8.41±1.62 | 8.39±1.59 | — | 3 | ② | Random |
| Liu2019-2 | NA | 74/74 | 74/74 | 38/36 | 36/38 | — | CTX8-12mg/(kg·d)+PRED1-2mg/kg+DIPY+VC | MMF15-20mg/kg+PRED1-2mg/kg+DIPY+VC | — | 9.08±1.92 | 9.07±2.93 | — | 12 | ② | Random |
| Du2010 | 24hUpro > 50mg/（kg·d） | 25/40 | 38/27 | 14/11 | 24/16 | — | CTX10mg/(kg·d)+PRED2mg/(kg·d)+UFH+DIPY+VD+Ca | MMF20-25mg/(kg·d)+PRED2mg/(kg·d)+UFH+DIPY+VD+Ca | — | 6.9±2.2 | 7.9±2.9 | — | 12 | ①③ | Random |
| Jiang2016 | 24hUpro≥ 50mg/（kg·d） | 24/31 | 37/18 | 17/7 | 20/11 | — | CTX8-12mg/(kg·d)+PRED 1.5-2mg/(kg·d) | MMF15-20/(kg·d)mg+PRED1mg/(kg·d) | — | 8. 5±2. 9 | 8. 0±2. 2 | — | 12 | ①③ | Random |
| Li2019 | CMA2001 | 70/70 | 84/56 | 43/27 | 41/29 | — | CTX10mg/(kg·d)+PRED1mg/(kg·d) | MMF20mg/(kg·d)+PRED0.5mg/(kg·d) | — | 8.81±2.38 | 8.62±2.47 | — | 6 | ①② | Random Number Table |
| Li2021 | 24hUpro > 50mg/（kg·d） | 42/42 | 60/24 | 31/11 | 29/13 | — | CTX+GCs | MMF30-40mg/d+GCs | — | 8.31±1.62 | 7.31±1.41 | — | unclear | ① | Random Number Table |
| Li2018 | NA | 36/36 | 39/33 | 20/16 | 19/17 | — | Hydrocort.20-40mg/d | MMF2g/d+Hydrocort.20-40mg/d | — | 8.5±3.2 | 8.5±2.2 | — | 18 | ②⑥⑦ | Random Number Table |
| Huang2018 | NA | 35/35 | 35/35 | 21/14 | 24/11 | — | PRED10-20mg/d+DIPY75-150mg/d+VC+Ca | MMF20-30mg/d+PRED10-20mg/d+DIPY75-150mg/d+VC+Ca | — | 7.45±1.24 | 7.44±1.22 | — | 6 | ② | lottery method |
| He2011 | CMA2009 | 46/46 | 59/33 | 30/16 | 29/17 | — | PRED1.5-2mg/(kg·d)+DIPY3-5mg/(kg·d) | CTX8-12mg/kg+PRED1.5-2mg/(kg·d)+DIPY3-5mg/(kg·d) | — | 10.5±2.3 | 10.2±2.5 | — | 1 | ② | Random Number Table |
| A2016 | CMA2009 | 19/23 | 22/20 | 9/10 | 13/10 | — | PRED2mg/(kg·d)+DIPY1-2mg/(kg·d) | CTX10mg/kg+PRED2mg/(kg·d)+DIPY1-2mg/(kg·d) | — | 7. 9±2. 1 | 8. 2±2. 4 | — | unclear | ①③④⑤ | Random Number Table |
| Liu2008 | NA | 20/20 | 23/17 | 12/8 | 11/9 | — | PRED1.5-2mg/(kg·d)+DIPY3-5mg/(kg·d) | CTX12mg/(kg·d)+PRED1.5-2mg/(kg·d)+DIPY3-5mg/(kg·d) | — | 8.0 | 7.8 | — | unclear | ①③ | Random |
| Bai2021 | 24hUpro > 150mg/d | 43/43 | 42/44 | 22/21 | 20/23 | — | MP15-30mg/(kg·d) | CsA150mg/d+MP15-30mg/(kg·d) | — | 7.15±3.05 | 7.08±2.62 | — | 6 | ①② | Random Number Table |
| Liao2016 | NA | 42/43 | 41/44 | 20/22 | 21/22 | — | MP1.6mg/(kg·d)+DIPY3mg/(kg·d)+UFH1mg/(kg·d) | CTX10mg/(kg·d)+MP1.6mg/(kg·d)+VC30mg/(kg·d)+DIPY3mg/(kg·d)+UFH1mg/(kg·d) | — | 7.15±1.37 | 7.19±1.35 | — | 7 | ① | lottery method |
| Lu2012 | NA | 12/18 | 20/10 | 8/4 | 12/6 | — | MP1.6mg/(kg·d)+DIPY3mg/(kg·d)+UFH1mg/(kg·d) | CTX8-10mg/(kg·d)+MP1.6mg/(kg·d)+VC30mg/(kg·d)+DIPY3mg/(kg·d)+UFH1mg/(kg·d) | — | 8. 3±2. 6 | 7. 8±3. 1 | — | unclear | ① | Random |
| Zhang2005 | CMA2001 | 15/19 | 22/12 | 10/5 | 12/7 | — | PRED2mg/(kg·d)+DIPY3mg/(kg·d)+UFH1-2mg/(kg·d) | CTX8-10mg/(kg·d)+PRED1mg/(kg·d)+VC50mg/kg+DIPY3mg/(kg·d)+UFH1-2mg/(kg·d) | — | 7±2 | 7±4 | — | unclear | ① | Random |
| Mai2009 | NA | 20/21 | 24/17 | 11/9 | 13/8 | — | PRED1-2mg/(kg·d) | MMF20-25mg/(kg·d)+PRED1-2mg/(kg·d) | — | 9.7±4.1 | 9.3±3.5 | — | 12 | ②⑥⑦ | Random |
| Mao2005 | CMA2001 | 20/20 | 23/17 | 12/8 | 11/9 | — | PRED1.5-2  mg/(kg·d)+DIPY1-2g/(kg·d) | CTX8-10mg/(kg·d)+  PRED1.5-2mg/(kg·d)+  DIPY1-2g/(kg·d) | — | unclear | unclear | — | 4 | ①②③ | Random |
| Shi2021 | CMA2016 | 36/36 | 41/31 | 20/16 | 21/15 | — | PRED0.5-1.0mg/(kg·d)+CTX10mg/(kg·d) | PRED0.5-1.0mg/(kg·d)+MMF7.5-15mg/(kg·d) | — | 7.2±1.2 | 7.3±1.2 | — | 6 | ①②④⑥⑦ | Random |
| Sun2015 | CMA2001 | 41/42 | 51/32 | 26/15 | 25/17 | — | CT | CT+TWP1mg/(kg·d) | — | 8.5±2.2 | 8.6±2.4 | — | 3 | ①② | Random Number Table |
| Wang2011 | CMA2009 | 23/23 | 26/20 | — | — | — | PRED1.0-2.0mg/(kg·d)+DIPY | PRED1.0-2.0mg/(kg·d)+DIPY+MMF20mg/(kg·d) | — | — | — | — | 9 | ①② | Random |
| Wang2020 | NA | 32/32 | 39/25 | 19/13 | 20/12 | — | PRED2.0mg/(kg·d) | PRED2.0mg/(kg·d)+CTX12mg/(kg·d) | — | 7.5±2.9 | 7.9±2.7 | — | 6 | ①③④ | Random Number Table |
| Wang2008 | CMA2003 | 30/30 | 35/25 | 18/12 | 17/13 | — | DIPY5-10mg/(kg·d)+VC40-80mg/(kg·d)+ TROXE4-8mg/(kg·d)+ CPA0.35mg/(kg·d)+CIM12-16mg/(kg·d)+MP15-20mg/(kg·d)+MMF20mg/(kg·d)+HC1-2mg/(kg·d) | DIPY5-10mg/(kg·d)+VC40-80mg/(kg·d)+ TROXE4-8mg/(kg·d)+ CPA0.35mg/(kg·d)+CIM12-16mg/(kg·d)+MP15-20mg/(kg·d)+CTX10mg/(kg·d)+LMWH1-2mg/(kg·d) | — | 9.8±4.3 | 10.1±4.2 | — | 12 | ①②③④ | Random |
| Wang2009 | CMA2001 | 19/18 | 21/16 | 11/8 | 10/8 | — | PRED0.5-1.0mg/(kg·d)+LEF1mg/(kg·d) | PRED0.5-1.0mg/(kg·d)+CTX8-12mg/(kg·d) | — | 10.5±4.2 | 11.2±3.1 | — | 6 | ①② | Random |
| Wu2015 | 24hUpro > 50mg/（kg·d） | 24/31 | 37/18 | 17/7 | 20/11 | — | PRED1.5-2.0mg/(kg·d)+CTX8-12mg/(kg·d) | PRED1.0mg/(kg·d)+MMF15-29PRED1.5-2.0mg/(kg·d) | — | 8.5±2.9 | 8.0±2.2 | — | 4 | ①②③ | Random |
| Xu2009 | CMA2001 | 10/9 | 12/7 | — | — | — | PRED1.0-1.5mg/(kg·d) | PRED1mg/(kg·d)+MMF20mg/(kg·d) | — | — | — | — | unclear | ①③ | Random |
| Yang2013 | NA | 21/21 | 23/19 | 11/10 | 12/9 | — | PRED0.5-1.0mg/(kg·d)+DIPY3-5mg/(kg·d)+CTX8-12 | PRED0.5-1.0mg/(kg·d)+DIPY3-5mg/(kg·d)+MMF20-25mg/(kg·d) | — | 13.2±3.5 | 12.5±2.6 | — | 9 | ① | Random Number Table |
| Ye2024 | CMA2009 | 44/44 | 45/43 | 23/21 | 22/22 | — | MP20mg/(kg·d)+CTX3mg/(kg·d) | MP20mg/(kg·d)+TAC0.1mg/(kg·d)+ | — | 7.04±0.61 | 7.75±0.56 | — | 1 | ②④⑤ | Random |
| Yu2007 | CMA2001 | 18/19 | 21/16 | 10/8 | 11/8 | — | PRED0.5-1.0mg/(kg·d)+ACEI5-10mg/d+ARB25-50mg/d+DIPY3-5mg/(kg·d)+CTX8-12mg/(kg·d) | PRED0.5-1.0mg/(kg·d)+ACEI5-10mg/d+ARB25-50mg/d+DIPY3-5mg/(kg·d)+MMF20-25mg/(kg·d) | — | 11.2±3.1 | 10.5±4.2 | — | unclear | ①② | Random |
| Yu2019 | CMA2009 | 51/51 | 49/53 | 23/28 | 26/25 | — | DIPY75mg/d+VC300mg+PRED0.5mg/(kg·d)+CTX750 mg·m^-2^ | PRED0.5mg/(kg·d)+TAC0.05mg/(kg·d) | — | 6.74±1.08 | 6.87±1.13 | — | 3 | ①②④⑤ | Random Number Table |
| Zhang2015 | CMA2009 | 44/46 | 53/37 | 25/19 | 28/18 | — | PRED2mg/(kg·d)+CTX8mg/(kg·d) | PRED1mg/(kg·d)+MMF15mg/(kg·d) | — | 6.5±2.8 | 7.6±2.5 | — | 3 | ②③ | Random Number Table |
| Zhang2022 | CMA2016 | 35/35/35 | 60/45 | 20/15 | 19/16 | 21/14 | PRED1.5mg/(kg·d)+CTX8-12mg/(kg·d) | PRED1.5mg/(kg·d)+MMF15mg/(kg·d) | — | 8.54±2.04 | 8.71±2.10 | 9.08±2.04 | 3 | ② | Random |
| Zhang2019 | 24hUpro > 50mg/（kg·d) | 20/20 | — | — | — | — | PRED1.5-2mg/(kg·d)+CTX8-12mg/(kg·d) | PRED1.5-2mg/(kg·d)+MMF20-30mg/(kg·d) | — | — | — | — | unclear | ② | Lottery method |
| Zou2015 | NA | 50/50 | 56/44 | 30/20 | 26/24 | — | PRED1.5-2mg/(kg·d)+DIPY3-5mg/(kg·d) | CTX8-12mg/(kg·d) | — | 10.5±2.3 | 10.2±2.5 | — | unclear | ② | Random |
| Zou2018 | NA | 30/30 | 29/31 | 15/15 | 14/16 | — | PRED2mg/(kg·d) | PRED2mg/(kg·d)+CTX12mg/(kg·d) | — | 6.4±1.9 | 6.6±1.5 | — | 6 | ①③④⑤ | Random Number Table |
| Peng2019 | NA | 69/69 | 82/56 | 43/26 | 39/30 | — | MP0.25-0.5g/d+PRED1.5mg/(kg·d)+MMF0.75-1.0 g/d | MP0.25-0.5g/d+PRED1.5mg/(kg·d)+TAC0.05-0.1mg/(kg·d) | — | 7.42±0.88 | 7.35±0.83 | — | 6 | ④⑤ | Random Number Table |
| Qiao2018 | 24hUpro > 50mg/（kg·d) | 42/42 | 60/24 | 31/11 | 29/13 | — | CTX8-12mg/(kg·d)+PRED1mg/(kg·d) | MMF15-20mg/(kg·d)+PRED1mg/(kg·d) | — | 8.3±1.6 | 7.5±1.7 | — | unclear | ②④⑥⑦ | Random Number Table |
| Song2017 | NA | 32/32 | 35/29 | 18/14 | 17/15 | — | UFH1mg/kg+MP1.6mg/kg | UFH1mg/kg+CTX8-20mg/kg | — | 8.5±1.2 | 8.5±1.4 | — | unclear | ① | Random Number Table |
| Yin2023 | CMA2016 | 21/21 | 23/19 | 12/9 | 11/10 | — | PRED0.5-1mg/(kg·d)+CTX8-12mg/(kg·d) | PRED1mg/(kg·d)+MMF15-20mg/(kg·d) | — | 7.52±1.44 | 7.62±1.21 | — | 6 | ①②④⑤ | Random Number Table |
| Yuan2019 | NA | 42/42 | 43/41 | 24/18 | 19/23 | — | CTX8-12mg/(kg·d)+PRED0.5-1mg/(kg·d) | MMF15-20mg/(kg·d)+PRED1mg/(kg·d) | — | 7.6±3.2 | 7.3±2.6 | — | 6 | ①②④⑤ | Random Number Table |
| Zhang2021 | 24hUpro > 50mg/（kg·d) | 83/87 | 90/80 | 47/36 | 43/44 | — | PRED1.5–2 µg/kg/d+CTX500–750 m^2^/kg | TAC0.1-0.15mg/(kg·d) | — | 8.74±2.79 | 8.52±3.05 | — | 6 | ①②④⑤ | Random |
| Zhang2021-2 | 24hUpro > 50mg/（kg·d) | 87/85 | 86/86 | 43/44 | 43/42 | — | TAC0.1-0.15mg/(kg·d) | TAC0.1-0.15mg/(kg·d)+TWP2mg/(kg·d) | — | 8.52±3.05 | 8.69±3.01 | — | 6 | ①②④⑤ | Random |
| Sun2016 | CMA2000 | 50/50 | 54/46 | 25/25 | 29/21 | — | DIPY3mg/(kg·d)+UFH1mg/(kg·d)+VC+VE0.3mg/d+MP15-30mg/(kg·d) | DIPY3mg/(kg·d)+UFH1mg/(kg·d)+VC+VE0.3mg/d+MP15-30mg/(kg·d)+CTX8-10mg/(kg·d) | — | 9.2±1.2 | 9.4±1.4 | — | 6 | ① | Random |
| Lu2020 | CMA2016 | 23/23 | 27/19 | 14/9 | 13/10 | — | PRED2mg/(kg·d) | PRED2mg/(kg·d)+CTX12mg/(kg·d) | — | 6.85±1.32 | 7.36±1.08 | — | 6 | ①⑤ | Random |
| Chen2013 | NA | 12/18 | — | — | — | — | PRED2mg/(kg·d)+DIPY1-2mg/(kg·d) | PRED2mg/(kg·d)+DIPY1-2mg/(kg·d)+CTX8-12mg/(kg·d) | — | 8.7±2.6 | 8.5±2.8 | — | unclear | ①③⑤ | Random |

Note: CT:Conventional Therapy; PRED:Prednisone; MP:Methylprednisolone; DIPY:Dipyridamole; UFH:Unfractionated Heparin; HC:Heparin Calcium; LMWH:Low Molecular Weight Heparin ;TROXE:Troxerutin; CPA :Chlorpheniramine; CIM:Cimetidine; CaD3:Calcium Carbonate and Vitamin D3 Tablets; Ca:Calcium Supplement; C.R.:Compound Rutin Tablets; LOR:Loratadine; DEX:Dexamethasone; GCs:Glucocorticoids; Hydrocort. :Hydrocortisone; ACEI:Angiotensin-Converting Enzyme Inhibitor; ARB:Angiotensin II Receptor Blocker; VC:Vitamin C; VE:Vitamin E (Tocopherol)

①24-Hour Urine Protein Quantification (24hU-Pro);②Incidence of adverse events (AE incidence);③Serum albumin(ALB);④ Serum creatinine (SCr);⑤:Blood urea nitrogen (BUN);⑥Time To Proteinuria Resolution (TTPR) ⑦Time To Hematuria Resolution (TTHR)

**Supplementary Table S3 –** Results of network Meta-Analysis (24hU-Pro).

| MMF+CT |  |  |  |  |  |  |  |  |
| --- | --- | --- | --- | --- | --- | --- | --- | --- |
| -0.01 (-1.37,1.35) | LEF+CT |  |  |  |  |  |  |  |
| -0.24 (-1.10,0.63) | -0.23 (-1.81,1.35) | TAC+CT |  |  |  |  |  |  |
| -0.26 (-2.03,1.52) | -0.25 (-2.44,1.95) | -0.02 (-1.97,1.93) | TAC+TWP |  |  |  |  |  |
| -0.29 (-1.57,0.99) | -0.28 (-2.09,1.54) | -0.05 (-1.56,1.46) | -0.03 (-1.26,1.20) | TAC |  |  |  |  |
| -0.35 (-0.69,-0.01) | -0.33 (-1.66,0.99) | -0.11 (-0.98,0.76) | -0.09 (-1.83,1.66) | -0.06 (-1.29,1.17) | CTX+CT |  |  |  |
| -1.20 (-2.50,0.11) | -1.19 (-2.99,0.62) | -0.96 (-2.49,0.57) | -0.94 (-3.10,1.22) | -0.91 (-2.68,0.87) | -0.85 (-2.13,0.43) | CsA+CT |  |  |
| -1.43 (-2.27,-0.59) | -1.42 (-2.92,0.08) | -1.19 (-2.35,-0.03) | -1.17 (-3.09,0.74) | -1.14 (-2.61,0.32) | -1.08 (-1.88,-0.29) | -0.23 (-1.64,1.18) | TWP+CT |  |
| -1.60 (-2.06,-1.14) | -1.59 (-2.91,-0.26) | -1.36 (-2.28,-0.44) | -1.34 (-3.12,0.44) | -1.31 (-2.60,-0.02) | -1.25 (-1.62,-0.88) | -0.40 (-1.62,0.82) | -0.17 (-0.87,0.54) | CT |

Results from the network meta-analysis (mixed [network] and indirect comparisons) are presented in the lower left triangle.Deeper background color means statistically significant.Note:CT:Conventional Therapy.MMF:Mycophenolate Mofetil; LEF:Leflunomide; TAC:Tacrolimus; TWP:Tripterygium Wilfordii Polyglycosides; CTX:Cyclophosphamide;CsA:Cyclosporine A.

**Supplementary Table S4 –** Results of network Meta-Analysis (ALB).

| MMF+CT |  |  |  |  |
| --- | --- | --- | --- | --- |
| 0.23 (-0.50 ,0.96) | TAC+CT |  |  |  |
| 0.25 (-0.61,1.11) | 0.02 (-1.11,1.15) | LEF+CT |  |  |
| 0.29 (0.03,0.54) | 0.05 (-0.72,0.83) | 0.03 (-0.80,0.86) | CTX+CT |  |
| 1.07 (0.69,1.45) | 0.84 (0.01,1.66) | 0.82 (0.04,1.59) | 0.78 (0.48,1.08) | CT |

Results from the network meta-analysis (mixed [network] and indirect comparisons) are presented in the lower left triangle.Deeper background color means statistically significant.Note:CT:Conventional Therapy.MMF:Mycophenolate Mofetil; LEF:Leflunomide; TAC:Tacrolimus; CTX:Cyclophosphamide.

**Supplementary Table S5 –** Results of network Meta-Analysis (SCr).

| TAC+CT |  |  |  |  |  |  |
| --- | --- | --- | --- | --- | --- | --- |
| -1.00 (-2.86,0.86) | LEF+CT |  |  |  |  |  |
| -1.13 (-2.02,-0.25) | -0.13 (-1.89,1.62) | MMF+CT |  |  |  |  |
| -1.63 (-3.22,-0.04) | -0.63 (-2.78,1.52) | -0.49 (-1.96,0.97) | TAC |  |  |  |
| -1.90 (-2.73,-1.07) | -0.90 (-2.57,0.76) | -0.77 (-1.32,-0.22) | -0.27 (-1.63,1.08) | CTX+CT |  |  |
| -2.40 (-4.49,-0.31) | -1.40 (-3.94,1.14) | -1.27 (-3.26,0.73) | -0.77 (-2.13,0.59) | -0.50 (-2.42,1.42) | TAC+TWP |  |
| -2.49 (-3.67,-1.32) | -1.49 (-2.94,-0.05) | -1.36 (-2.35,-0.37) | -0.87 (-2.46,0.72) | -0.59 (-1.42,0.23) | -0.10 (-2.19,2.00) | CT |

Results from the network meta-analysis (mixed [network] and indirect comparisons) are presented in the lower left triangle.Deeper background color means statistically significant.Note:CT:Conventional Therapy.MMF:Mycophenolate Mofetil; LEF:Leflunomide; TAC:Tacrolimus; CTX:Cyclophosphamide; TWP:Tripterygium Wilfordii Polyglycosides.

**Supplementary Table S6 –** Results of network Meta-Analysis (BUN).

| MMF+CT |  |  |  |  |  |  |
| --- | --- | --- | --- | --- | --- | --- |
| -0.69 (-2.70,1.32) | TAC+CT |  |  |  |  |  |
| -1.70 (-5.04,1.63) | -1.01 (-4.27,2.24) | TAC |  |  |  |  |
| -1.83 (-6.17,2.52) | -1.13 (-5.42,3.15) | -0.12 (-2.91,2.66) | TAC+TWP |  |  |  |
| -1.90 (-3.75,-0.06) | -1.21 (-2.92,0.49) | -0.20 (-2.97,2.58) | -0.08 (-4.01,3.85) | CTX+CT |  |  |
| -2.26 (-5.89,1.37) | -1.57 (-5.13,1.99) | -0.56 (-4.74,3.63) | -0.44 (-5.46,4.59) | -0.36 (-3.49,2.78) | LEF+CT |  |
| -2.53 (-4.81,-0.25) | -1.84 (-4.00,0.32) | -0.83 (-3.91,2.26) | -0.71 (-4.86,3.45) | -0.63 (-1.97,0.71) | -0.27 (-3.10,2.56) | CT |

Results from the network meta-analysis (mixed [network] and indirect comparisons) are presented in the lower left triangle.Deeper background color means statistically significant.Note:CT:Conventional Therapy.MMF:Mycophenolate Mofetil; LEF:Leflunomide; TAC:Tacrolimus; CTX:Cyclophosphamide; TWP:Tripterygium Wilfordii Polyglycosides.

**Supplementary Table S7 –** Results of network Meta-Analysis (TTPR).

| TWP+CT |  |  |  |
| --- | --- | --- | --- |
| -5.79 (-8.93,-2.65) | MMF+CT |  |  |
| -9.56 (-11.28,-7.83) | -3.77 (-6.41,-1.13) | CT |  |
| -13.89 (-17.41,-10.38) | -8.10 (-9.67,-6.53) | -4.33 (-7.44,-1.23) | CTX+CT |

Results from the network meta-analysis (mixed [network] and indirect comparisons) are presented in the lower left triangle.Deeper background color means statistically significant.Note:CT:Conventional Therapy.MMF:Mycophenolate Mofetil; CTX:Cyclophosphamide; TWP:Tripterygium Wilfordii Polyglycosides.

**Supplementary Table S8 –** Results of network Meta-Analysis (TTHR).

| TWP+CT |  |  |  |
| --- | --- | --- | --- |
| -22.27 (-48.42,3.88) | MMF+CT |  |  |
| -26.21 (-40.23,-12.19) | -3.94 (-26.01,18.13) | CT |  |
| -52.17 (-81.82,-22.53) | -29.90 (-43.93,-15.88) | -25.96 (-52.08,0.16) | CTX+CT |

Results from the network meta-analysis (mixed [network] and indirect comparisons) are presented in the lower left triangle.Deeper background color means statistically significant.Note:CT:Conventional Therapy.MMF:Mycophenolate Mofetil; CTX:Cyclophosphamide; TWP:Tripterygium Wilfordii Polyglycosides.

**Supplementary Table S9 –** Results of network Meta-Analysis (AE incidence).

| CsA+CT |  |  |  |  |  |  |  |  |  |
| --- | --- | --- | --- | --- | --- | --- | --- | --- | --- |
| 1.33 (0.11,16.01) | CTX |  |  |  |  |  |  |  |  |
| 0.61 (0.16,2.30) | 0.46 (0.05,4.17) | MMF+CT |  |  |  |  |  |  |  |
| 0.47 (0.11,1.93) | 0.35 (0.04,3.38) | 0.77 (0.38,1.54) | TAC+CT |  |  |  |  |  |  |
| 0.42 (0.08,2.27) | 0.31 (0.03,3.61) | 0.68 (0.23,1.99) | 0.89 (0.25,3.10) | LEF+CT |  |  |  |  |  |
| 0.35 (0.09,1.40) | 0.26 (0.03,2.49) | 0.58 (0.38,0.87) | 0.76 (0.35,1.61) | 0.85 (0.29,2.54) | TAC+TWP |  |  |  |  |
| 0.34 (0.09,1.29) | 0.25 (0.03,2.32) | 0.55 (0.27,1.13) | 0.72 (0.30,1.72) | 0.81 (0.22,2.91) | 0.95 (0.42,2.13) | TWP+CT |  |  |  |
| 0.33 (0.10,1.15) | 0.25 (0.03,2.16) | 0.55 (0.34,0.88) | 0.71 (0.36,1.43) | 0.80 (0.25,2.57) | 0.94 (0.52,1.73) | 0.99 (0.58,1.69) | CT |  |  |
| 0.27 (0.07,1.05) | 0.20 (0.02,1.89) | 0.45 (0.33,0.61) | 0.58 (0.29,1.18) | 0.66 (0.23,1.90) | 0.77 (0.60,1.00) | 0.81 (0.38,1.74) | 0.82 (0.47,1.41) | TAC |  |
| 0.22 (0.06,0.86) | 0.17 (0.02,1.55) | 0.37 (0.29,0.47) | 0.48 (0.24,0.95) | 0.54 (0.19,1.54) | 0.64 (0.46,0.88) | 0.67 (0.32,1.40) | 0.67 (0.40,1.12) | 0.82 (0.68,0.99) | CTX+CT |

Results from the network meta-analysis (mixed [network] and indirect comparisons) are presented in the lower left triangle.Deeper background color means statistically significant.Note:CT:Conventional Therapy.MMF:Mycophenolate Mofetil; LEF:Leflunomide; TAC:Tacrolimus; TWP:Tripterygium Wilfordii Polyglycosides; CTX:Cyclophosphamide;CsA:Cyclosporine A.

**Supplementary Table S10 –**Treatment rankings and SUCRA values from the network meta-analysis for each outcome.

| **Treatment** | **24h-UP** | **Rank** | **ALB** | **Rank** | **BUN** | **Rank** | **SCR** | **Rank** | **HDT** | **Rank** | **PDT** | **Rank** | **AE** | **Rank** |
| --- | --- | --- | --- | --- | --- | --- | --- | --- | --- | --- | --- | --- | --- | --- |
| CT | 8.6％ | 9 | 0.9％ | 5 | 22.4％ | 7 | 12.3％ | 7 | 44.7％ | 3 | 33.3％ | 3 | 36.6％ | 8 |
| CTX | — | — | — | — | — | — | — | — | — | — | — | — | 83.1％ | 2 |
| CTX+CT | 57.1％ | 6 | 48.6％ | 4 | 40.5％ | 5 | 35.1％ | 5 | 0.9％ | 4 | 0.1％ | 4 | 5％ | 10 |
| TAC | 61.6％ | 5 |  |  | 47％ | 3 | 49％ | 4 | — | — | — | — | 21.5％ | 9 |
| TAC+CT | 64.9％ | 3 | 57.5％ | 2 | 72.7％ | 2 | 97％ | 1 | — | — | — | — | 60.3％ | 4 |
| MMF+CT | 80.4％ | 1 | 86％ | 1 | 87.3％ | 1 | 68％ | 3 | 56.1％ | 2 | 66.6％ | 2 | 77.4％ | 3 |
| LEF+CT | 73.5％ | 2 | 56.9％ | 3 | 36.1％ | 6 | 68.5％ | 2 | — | — | — | — | 50.4％ | 5 |
| TWP+CT | 15.8％ | 8 | — | — | — | — | — | — | 98.3％ | 1 | 100％ | 1 | 37.1％ | 7 |
| CsA+CT | 26.3％ | 7 | — | — | — | — | — | — | — | — | — | — | 85.2％ | 1 |
| TAC+TWP | 61.9％ | 4 | — | — | 44.1％ | 4 | 20.1％ | 6 | — | — | — | — | 43.5％ | 6 |

**Supplementary Figure S1 –** Risk of bias assessment included in the study
